# Abstracts from the 7th International Conference for Healthcare and Medical Students (ICHAMS): Dublin, Ireland. 16-18 February 2018

**DOI:** 10.1186/s12919-018-0157-2

**Published:** 2018-11-06

## A1 Factors contributing to poor medical adherence in patients after acute coronary syndrome

### Elizabete Terauda^1^, Karlis Trusinskis^2^, Alberts Berzins^3^

#### ^1^Faculty of Medicine, Riga Stradins University, Riga, Latvia; ^2^Pauls Stradins Clinical University Hospital, Latvian Centre of Cardiology, Riga, Latvia

**Introduction**

Adherence to secondary prevention medications following acute coronary syndrome (ACS) is low, standing around 40–75% by various estimates. Our aim was to assess patient adherence to pharmacotherapy and analyse reasons for non-compliance.

**Methods**

A prospective study includes patients with ACS, hospitalized in Pauls Stradins Clinical University Hospital. During the period from September 2016 until March 2017 78 patients were interviewed. Data from medical records were collected. 6-month follow-ups were conducted by telephone interview. The obtained data were analysed by IBM SPSS.

**Results**

A 6-month follow-up was conducted from 67 patients. 6 patients (7.7%) had died and 5 (6.4%) were lost to follow-up. At discharge for 63 patients (80.7%) dual antiplatelet therapy (DAPT) was recommended, 65.7% (n=44) use DAPT after 6 months. Statin therapy was recommended to 97.4% (n=76) of patients, at 6-month follow-up 85.0% (n=57) take statins. Atorvastatin 80mg was recommended to 76.9% (n=60), at follow-up 52.2% (n=35) take the same dose. Main reasons for non-adherence to dual antiplatelet therapy were reimbursement discontinuation (13.4%) and adverse effects (5.9%). In 23.8% of patients changes in antiplatelet therapy were attributed to decision of cardiologist or general practitioner. 19.4% of patients lack motivation to take statins at recommended dose. In 10.4% of cases general practitioner or cardiologist changed statin therapy.

**Discussion**

For statin therapy the main reason for non-compliance was lack of motivation, for antiplatelet therapy - discontinuation of reimbursement and adverse side effects. Adherence to pharmacotherapy at 6-month follow-up was poor and was influenced by lack of understanding of the aim of pharmacotherapy as well as deficient reimbursement policy.

## A2 The impact of interruptions on pediatric resuscitation team performance

### Carol Rizkalla^1^, Elaine Gilfoyle^2^

#### ^1^Medicine, Royal College of Surgeons in Ireland, Dublin, Ireland; ^2^PICU, Alberta Children's Hospital, Calgary, Canada

**Introduction**

Resuscitation is a complex and highly stressful clinical event. Team members need to be attentive to their environment at all times in order to quickly recognize and deal with changes in a patient’s status. Interruptions may adversely affect team performance and therefore patient outcomes. This study describes the impact of interruptions on pediatric resuscitation team performance.

**Methods**

This is an analysis of data previously collected during the Teams4Kids study. Resuscitation teams participated in a simulation-based team-training course. One of the video-recorded simulated scenarios involved a planned interruption at a key clinical transition point, where leaders were informed of a critical laboratory result, either by call or printout. Team behaviour was coded around this transition point. A validated scale was used to rate the severity of the interruption (1=no impact, 6=task must be repeated).

**Results**

The median time for initiating chest compressions and administering a shock decreased by over 30 seconds from PRE to POST team training, meeting the American Heart Association (AHA) guidelines. The interruption scale rating occurring most frequently in the PRE scenarios is a rating of 5, whereas in the POST scenarios it is a less severe rating of 3. When the leader is not involved in the analysis of paper results, the majority of the teams analyse the results within 30 seconds and meet the AHA guidelines.

**Discussion**

A thorough understanding of how teams deal with common interruptions will allow us to improve the training provided to teams in the future, which may translate into improved patient outcomes.

## A3 Clinicobiological, functional and cognitive-behavioral evaluation of the obese patients with eating disorders

### Ingrid Stafie^1^, Celina Stafie^2^, Stefana Luca^2^, Ecaterina Gramaticu^2^, Andrei Avadanei^2^

#### ^1^Medicine, “Grigore T.Popa” University of Medicine and Pharmacy, Iasi, Romania; ^2^Allergology and Clinical Immunology, “Grigore T.Popa” University of Medicine and Pharmacy, Iasi, Romania

**Introduction**

Eating disorders represent deviations from the rhythm and normal quantity of food of an individual and they are represented by anorexia and bulimia. Complications refer to serious hydroelectrolytic imbalance, metabolic, psychotic disorders and exitus. Anorexia and bulimia are triggered by a distress, which is considered to be associated with the “mourning period”: parents’ divorce or the loss of a family member.

**Methods**

The study focused on 82 patients with normal weight and/or with abdominal obesity, ambulatory care, Caucasians, aged 16 to 49 years old, IMC>28. The patients were studied between November 2015 and May 2017 and the ratio of women to men was 3/1. Clinical and biochemical parameters were evaluated to appreciate the cardiovascular and diabetes risk profile, neurological and cardio-respiratory functional investigations were performed, as well as the psychological evaluation (DSM-IV questionnaire. The final results have been interpreted statistically and cognitive-behavioral therapy has also been applied.

**Results**

The results obtained from the study cases have been divided based on the personality type. 85.2% of the patients have eating disorders and 26% present moderate and high cardiovascular risk in the next 10 years, women between 30 and 49 years old being the majority.

**Discussion**

A multidisciplinary approach can change eating behavior on a long term. The maximum effectiveness on cognitive behavioral therapy (CBT) could easily be observed on the group studies centered on self-affirmation development. Metabolic profile has also been improved, due to CBT.

## A4 Oxidative stress (OS) in drug using tuberculosis/HIV co-infected patients (DUs)

### Raja Raghupathy Rao Cherukuri^1^, Roman Mikhaylovich Yasinskiy^2^

#### ^1^Medicine, Zaporozhye State Medical University, Zaporozhye, Ukraine; ^2^Department of Phthisiology and Pulmonology, Zaporozhye State Medical University, Zaporozhye, Ukraine

**Introduction**

There are a variety of conditions that lead to OS in tuberculosis (TB), HIV and drug addicted patients, but there is no information about OS peculiarities in DUs. So, the aim of our study is to determine OS in DUs.

**Methods**

A prospective study of 54 patients with TB and HIV co-infection was done. They were divided into 2 groups. Group A consisted of 17 patients who are drug users and Group B consisted of 37 patients who are not drug users. Group C consisted of 32 healthy individuals. Blood indices for OS markers were analyzed in all of them. Statistical analysis was performed using Statistica6.0 software.

**Results**

Protein peroxidation markers, Aldehyde-Phenylhydrazone and Ketone-Phenylhydrazone were higher in A and B than in C (4.42±0.25 pA-C<0.01, 5.30±0.44, pB-C<0.001 vs. 3.81±0.09; 2.82±0.17, pA-C<0.01, 3.37±0.22, pB-C<0.001 vs. 2.32±0.09 optical density/g protein). Intermediate Mass Molecules (IMM) were higher in A and B (0.28±0.02, pA-C<0.001; 0.24±0.01, pB-C<0.001) when compared with C(0.13±0.004 units). Catalase activity (Cat) in A(3.01± 0.55; pA-C<0.05) and B(2.99±0.39; pB-C<0.01) was lower than in C(4.49±0.37 mcat/mg/minute). Superoxide Dismutase activity (SOD) was significantly increased in A(10.86±1.83, pA-C<0.001 and pA-B<0.001) when compared to B(4.47±0.75) and C(2.94±0.61 units/protein mg). Glutathione Peroxidase(GP) in A and B(12.85±5.7 and 11.67±1.4, pB-C<0.001) is lesser than in C(19.9±1.8 IU/g Hb).

**Discussion**

There is elevation of OS markers and reduction of Cat and GP levels in all TB/HIV co-infected patients. But SOD elevation in group A with decreased levels of Cat and GP has led to increased oxidative stress in DUs.

## A5 Comparison of microbiological characteristics of complicated influenza virus A(H1N1) pdm09 in the city of Smolensk in 2009 and 2016

### Anna Mikheeva, Olga Azovskova

#### Department of Microbiology, Smolensk State Medical University, Smolensk, Russia

**Introduction**

A new subtype of the influenza virus (A/H1N1/pdm09) caused pandemics in 2009 and 2016. A large number of complications such as viral and bacterial pneumonia determined the severity of the disease, the lethal outcome. The aim of the study was to ascertain etiology of the pneumonia in patients who died of the flu caused by virus H1N1/pdm09 in 2009 and 2016.

**Methods**

Tissue samples were investigated by direct microscopic and bacteriological methods. The inoculation of the material was performed on Brain Heart Infusion agar, Egg-yolk Salt Agar, Selective Enterococcus Agar, MacConkey agar, Sabouraud agar. Identification of selected pure cultures, determination of antibiotic sensitivity was performed with standard test kits.

**Results**

12 cases in 2009 and 11 cases in 2016 were analyzed. The clinical diagnosis of “flu” was confirmed antemortem or postmortem in all cases. In 2009 83%of cases were isolated Staphylococcus aureus. In 17% of cases the bacterial or fungal microflora were not revealed. In 2016 it was selected strains of Acinetobacter baumanii (55% of cases) and its association with Klebsiella pneumoniae (18% of cases). All isolated strains had developed antibiotic resistance. In 18% of cases it was ascertained strains of Staphylococcus aureus. In 9% of cases etiologically significant microorganisms were not detected.

**Discussion**

Thus, the occurrence of pneumonia with a fatal outcome in influenza in 2009 and 2016 was due to the accession of bacterial flora most often. Antibiotic resistance indicates nosocomial origin of the microorganisms. In 2009 the pneumonia was caused by Staphylococcus aureus; in 2016 – by Acinetobacter baumanii and Klebsiella pneumoniae mainly.

## A6 Analysis of objective reasons and human factors contribution to the ineffectiveness of stroke treatment

### Nikita Maslov, Marina Agafonova

#### Neurology and Neurosurgery, Smolensk State Medical University, Smolensk, Russia

**Introduction**

The mortality from cardiovascular diseases leads the worldwide mortality in recent years. This index was 48.7% all causes of death in the Russian Federation in 2016. It continues to be high, significantly exceeding the rate in the UK - 29%. The aim of the study was to analyze the features of the medical care provision to the fatal stroke patients in the region of Smolensk city in 2016-2017.

**Methods**

The analysis of the annual reports of the stroke treatment units in Smolensk region, stroke patients’ databases was performed using the Microsoft Excel 2007 software.

**Results**

An ischemic stroke was the cause of death in 72.94% cases (in 12.7% a stroke had the cardioembolic genesis); 25.46% – intracerebral hemorrhage; and only in 1.6% - subarachnoid hemorrhage. The average age at death was 72.51±12.62 years. The analysis of the concomitant conditions of the lethal stroke cases showed that 44.17% patients had atrial fibrillation, 21.74% - diabetes mellitus, 93.74% - arterial hypertension, which caused such a ratio of ischemic strokes to hemorrhagic as 3:1, not 5:1 as in Russia. Low rates of ischemic stroke patients’ delivery to the hospitals within the therapeutic regimen were identified (17.63% of the total number of patients with ischemic stroke).

**Discussion**

Thus, the morbidity and the mortality related to the stroke in Smolensk region significantly exceed the indexes for Russia and Western Europe. It is necessary to recommend strengthening of social advertising of risk factors and intensification of an outpatient control for primary and secondary prevention of stroke.

## A7 Are Slc4a4 ion transporters present on the interstitial cells of Cajal within the mouse small bowel?

### Pavandeep Takhar, Simon Gibbons, Gianrico Farrugia, Seth Eisenman

#### Enteric Neuroscience Program, Department of Physiology and Biomedical Engineering, Mayo Clinic, Rochester MN, United States of America

**Introduction**

Interstitial cells of Cajal (ICC) are a major component of the pace making syncytium of the bowel, and an integral element in normal gastrointestinal motility. The mechanism by which ICC produce electrical slow waves, the pacemaker potential in the bowel is poorly understood. In this study, it was hypothesized that a bicarbonate ion transporter called Slc4a4 plays a role in pacemaker function of ICC. The aim of this experiment was to determine whether the Slc4a4 ion transporter is present within a subset of pacemaker ICC.

**Methods**

Antibodies directed against Slc4a4 and the receptor tyrosine kinase kit (a marker of ICC) were added to sections of mouse jejunum. Specific immunolabeling was detected using fluorochrome bound secondary antibodies. This was visualized by epifluorescence and laser-scanning confocal microscopy.

**Results**

Fluorescent labeling was observed indicating slc4a4-immunoreactivity in ICC that generate the electrical slow wave of the myenteric region of the mouse small intestine. ICC that do not generate electrical slow waves, in the deep muscular plexus from the same tissue were Kit positive but Slc4a4 negative. Immuno-reactivity for Slc4a4 was not observed in intestinal tissues from a Slc4a4 knock-out mouse - validating the specificity of the Slc4a4 antiserum.

**Discussion**

The results demonstrated expression of Slc4a4 ion transporters in a subset of ICC in mouse jejunum. The next step would be to characterize which of the several functional variants of Slc4a4 are expressed in pacemaker ICC and to repeat this experiment in human tissue, to further establish this relationship and understand its clinical significance.

## A8 Significance of P. aeruginosa colonization to pulmonary function, hospital and ambulatory admission rates in children with cystic fibrosis

### Akvile Smigelskyte, Violeta Radziuniene

#### Faculty of Medicine, Vilnius University, Vilnius, Lithuania

**Introduction**

Cystic fibrosis (CF) is an inherited disorder that causes severe damage to the lungs, digestive system and other organs. Pulmonary disease or infection could accelerate CF condition. Therefore, it is important to research their interactions and preserve pulmonary function as long as possible. The purpose of this study is to identify relationships between pulmonary function and P.aeruginosa (PsA) colonization in children with CF.

**Methods**

22 patients 5-18 years old with CF from ‘Vilnius City Clinical Hospital‘s Clinic of Children Diseases’ were enrolled in this study from January 2015 to October 2017. The pulmonary function was measured with spirometry test during ambulatory care visits and hospitalizations. Spirometry measurements included forced volume vital capacity (FVC) and forced expiratory volume in one second (FEV1). The information about PsA colonization, hospital and ambulatory admission rates and the number of bed-days in hospital were collected.

**Results**

Carriers of PsA are hospitalized longer than non-carriers respectively 14.6±3.785 days (p-value 0.016) and 10.05±3.052 (p-value 0.006). The carriers have also significantly (p-value 0.016) more ambulatory care visits to CF center (8.73±3.663) than the patients without PsA colonization (5.20±2.394). FVC and FEV1 at the time of hospitalization depend on PsA colonization (p-value 0.012 and 0.043), but there was no difference between groups during ambulatory care visits (p-value>0.05).

**Discussion**

The physicians should pay attention to PsA colonization because it may cause exacerbations of CF. Decreasing of lung function in children with CF might be slowed down if PsA infection will be diagnosed early and adequate treatment will be prescribed.

## A9 Profiling miRNA expression in patients with primary Sjogren’s syndrome

### Ameera Khan^1^, Conor Murphy^2^, Joan Ni Gabhann^3^

#### ^1^Medicine, Royal College of Surgeons in Ireland, Dublin, Ireland; ^2^Ophthalmology, Royal College of Surgeons in Ireland, Royal Victoria Eye and Ear Hospital, Dublin, Ireland; ^3^Molecular and Cellular Therapeutics, Royal College of Surgeons in Ireland, Dublin, Ireland

**Introduction**

Sjogren’s syndrome (SS) is an autoimmune inflammatory condition, with viral origins. This study looks at SS from the root inflammatory cause. Inflammation in SS arises due to lymphocytic cells infiltrating into exocrine glands. This is the result of unregulated cytokine production from the body’s defence cells. In SS, a viral infection can activate immune cells to produce interferon-alpha, enhancing the adaptive immune system in the body. Epithelial cells undergo apoptosis causing an autoimmune reaction from the body. MicroRNAs are an upcoming class of non-coding RNAs, which have an important role in the regulation of human genes, especially in autoimmune processes and diseases.

**Methods**

The aim of this research is to find a potentially therapeutic use of microRNA-focused treatment. By targeting significantly upregulated and downregulated miRNAs from SS patient’s blood sample, interesting miRNA can be examined. Genes looked at are homologous in rats, mice and humans, and have no isomers. Selection of the miRNAs was made by the use of bio-informatic techniques and the lab procedures in this study ranged from obtaining PBMC, cDNA, PCR and gel electrophoresis.

**Results**

The study focuses on the upregulated: miR-503-5p’s and its gene CREBL2. It also focuses on the downregulated: hsa-miR125b-1-3p, and hsa-miR-590-5p’s genes TGFBI and SCML2.

**Discussion**

Results from this study can be used to implement synthetic DNA sequences that mimic or antagonize the significantly upregulated or downregulated miRNAs and genes. The gel results show the most successful was miR-125-1-3p. It is elevated in ureteropelvic obstruction, indicating that it is active during inflammatory processes.

## A10 Pharmacokinetic analysis of midazolam and metabolites during pregnancy

### Joseph Saleh^1^, Wataru Ochiai^2^, Satoshi Kitaoka^2^, Jo Hatogai^2^, Nanako Niikura^2^, Satoru Miyazaki^2^

#### ^1^School of Pharmacy, Royal College of Surgeons in Ireland, Dublin, Ireland; ^2^Department of Clinical Pharmacokinetics, Hoshi University, Shinagawa, Tokyo, Japan

**Introduction**

Many women experience anxiety and insomnia during pregnancy, to rectify this benzodiazepines, in particular Midazolam (MDZ) are prescribed. Understanding the relationship between cytochrome P450 (CYP) enzymes in the liver and their ability to metabolise drugs is essential to quantify the role of MDZ in the development of embryo abnormalities. To analyse this relationship the main metabolites of MDZ, 1’-OH MDZ and 4-OH MDZ, must be quantified in the maternal plasma embryo. However, 4-OH MDZ is not metabolised to a significant amount in mice therefore it is not expected to be quantified in this experiment. For the mouse embryo, CYP3A16 (homologous to human CYP3A7) is the targeted isoform and for the mother, CYP3A11 (homologous to human CYP3A4/5) is the targeted isoform. Analysing this pharmacokinetic relationship was done using a combination of SPE and LC-MS.

**Methods**

The mice were weighed then their tails were marked to gather starting point data. They were then injected with MDZ (2.0 mg/kg) and caesarean sections were performed on the mice based on the following time frame (in minutes) from injection: 0, 5, 10, 15, 30, 45, 60, 90, 120, 150, 180, 240, 300. Maternal plasma and embryos were collected from the mice at these time frames. A SPE column was set up and the samples were loaded, then these samples were analysed using LC-MS. Once the chromatogram data was collected it was scrutinised using Napp (Numeric Analysis Program for Pharmacokinetics), a program which varied pharmacokinetic parameters.

**Results**

For the maternal plasma, the MDZ level is seen to be nearly completely depleted after 90 minutes. This deviates from the maternal plasma’s 1’-OH MDZ levels which peaks at 15 minutes. After the peak, the level decreases and plateaus by 120 minutes and decreases once more after 240 minutes. Different from the maternal plasma, the embryo’s MDZ levels peak at 15 minutes instead of time zero. The level doesn’t plateau for MDZ in the embryo until 180 minutes, unlike in maternal plasma. The peak level for 1’-OH MDZ in embryo occurs at 15 minutes where and follows a similar trend to the maternal plasma’s 1’-OH MDZ.

**Discussion**

The embryo lacks enzymes needed to metabolise MDZ into 1’-OH MDZ, which the mother has, so all of the systemic 1’-OH MDZ is received from the mother. The AUC ratio of 1’-OH MDZ: MDZ is roughly 1.4, suggesting that 1’-OH MDZ has a higher tendency to accumulate within the embryo, from the mother. This in turn could lead to malformation of the embryo as although 1’-OH MDZ has lower toxicity than MDZ, its levels are greater in the embryo therefore could increase incidence. Following from this data, it could then be suggested that use of benzodiazepines, particularly MDZ, could lead to malformations if used during pregnancy.

## A11 Glioblastomas developed in patients with a prior cancer: Epidemiological and pathological considerations on eleven cases

### Nicoleta Dumitrescu^1^, Gabriela Dumitrescu^2^, Alexandru Costache^3^, Bogdan Tarcau^3^, Sorana Anton^3^

#### ^1^Medicine, “Grigore T. Popa” University of Medicine and Pharmacy Iasi, Iasi, Romania; ^2^“Prof. Dr. N. Oblu” Pathology Laboratory, Emergency Clinical Hospital, Iasi, Romania; ^3^Medicine, “Grigore T. Popa” University of Medicine and Pharmacy Iasi, Iasi, Romania

**Introduction**

The risk factors that lead to the development of Central Nervous System (CNS) glioblastomas (GBs) are little known. The aim of our study was to identify the possible role of a prior cancer in the pathogenesis of a GB as there are few articles on this issue.

**Methods**

Records of patients with a prior non-CNS cancer and a GB treated in “Prof. Dr. N. Oblu” Emergency Clinical Hospital, Iasi, Romania between 2010-2016 were retrospectively reviewed. Data relating to epidemiological features and pathological characteristics were analysed.

**Results**

There were eleven patients having an average age with two peaks. The younger group (median age 48 years) had previously ductal mammary carcinomas (n=3) and intestinal adenocarcinomas (n=3). The elderly group (median age, 66.8 years) was diagnosed with prior squamous cell carcinomas (n=3) (located in penis, cervix, or larynx). GBs were diagnosed after a median interval of 91 months from the time of diagnosis of the non-CNS cancer (range, 49-218 months), but GBs was diagnosed after a two-fold lower period for mammary and digestive carcinomas compared to squamous carcinomas (68.6 months vs 118.8 months). Histologically, patients with prior breast and digestive carcinomas expressed the giant cell subtype of GB, but the others showed small cell GBs.

**Discussion**

GB may develop after non-CNS cancers expressing various locations and histological types. We suggest two etiopathogenic hypotheses of GB development in these cases: a genetic one, with significance for younger patients, and another one related to epigenetic factors, in the case of elderly patients.

## A12 Evaluation of different approaches in the application of vancomycin in intensive care unit patients

### Philipp Klocke^1^, Michael Tryba^2^, Damaris Meyn^2^

#### ^1^School of Medicine, University of Southampton, Southampton, United Kingdom; ^2^Department of Intensive Care Medicine, Gesundheit Nordhessen - Klinikum Kassel, Kassel, Germany

**Introduction**

Constant plateau levels in continuous vancomycin infusions (CVI) have recently been found to display similar clinical properties in terms of efficacy compared to intermittent dosing regimens (IVI). However, current scientific evidence shows no statistical superiority of either modality. This study aimed to investigate the efficacy of CVI using a new in-house dosing regimen.

**Methods**

ICU patients were retrospectively sought in a clinical audit using the hospital’s database and allocated towards a CVI or IVI group. Blood vancomycin serum concentrations and clinical data were extracted from files, compared and analysed using Fisher’s-exact and Student-t tests in SPSS.

**Results**

41 patients were included in this study {7 (CVI) vs. 34 (IVI)}. Both modalities achieved target concentrations while mean number of days to achieve target range was shorter in CVI compared to IVI (2.43±2.699 vs. 3.41±3.054; p=0.139). Number of days in target range was (CVI) 3.54±1.272 compared to (IVI) 2.74±2.079; p=0.385. Non-significant fluctuations around the target range were observed in both modalities. Mean amount of days below target range was 2.0±2.5 (CVI) vs. 3.59±2.583 (IVI); p= 0.134. However, mean amount of days above target range was 1.57±1.512 (CVI) vs. 0.5±0.862 (IVI); p= 0.058.

**Discussion**

Although not statistically significant, we could verify the benefits using CVI over IVI and show certain practical tendencies. We can assume that a more rapid achievement of target range and therein resulting stable plateau levels would improve vancomycin therapy. Larger trials using the same in-house regimen need to be carried out in near future to prove statistical significance.

## A13 Examining the association of cocaine and amphetamine-regulated transcript (CART) with Erα and proinflammatory cytokine expression in endometrial cancer

### Ibrahim Hayduran^1^, Darran O'Connor^2^, Sudipto Das^2^, Camille Hurley^2^, Brian Mooney^2^

#### ^1^School of Medicine, Royal College of Surgeons in Ireland, Dublin, Ireland; ^2^Molecular and Cellular Therapeutics, RCSI, Dublin, Ireland

**Introduction**

Identification of predictive biomarkers is extremely useful in improving therapeutic decisions. Cocaine and amphetamine-regulated transcript (CART) has been found to be a poor prognostic factor in estrogen receptor ER-positive, lymph node-negative tumors. Therefore, CART profiling may potentially allow personalization of therapy. Here, we further examine the impact of CART on ERα and inflammatory cytokines in endometrial cancer.

**Methods**

Total RNA was isolated from cell lines +/- CART alone, LPS (Lipopolysaccharide) alone, CART and LPS vehicles for two time points: 3 and 6 hours, using Trizol and reverse transcription according to the manufacturer’s instructions. qRT-PCR analysis was carried out for primers specific for IL1B, IL6, TNFα, ERα, PGR, PKIB and TFF1.

**Results**

Using qPCR we measured the effect of CART on mRNA expression of ERα, and downstream signaling partners of ERα including PgR (Progesterone receptor) and PKIB (Protein Kinase Inhibitor Beta). Data analysis confirmed that CART was associated with increased expression of ERα. It had a stronger effect than LPS in the samples incubated for 6 hours. Similarly, data revealed that CART also induced increased PgR, PKIB and TFF1 (ERα target gene) expression.

**Discussion**

It is important to mention that our findings are preliminary and further work including assessment of additional biological replicates are required to confirm these findings.

## A14 Are wait times to physician initial assessment (PIA) meeting the CTAS recommendations? A single-centre study at an urban community

### Megan Shum^1^, Leila Salehi^2^

#### ^1^Department of Emergency Medicine, Royal College of Surgeons in Ireland, Dublin, Ireland; ^2^University of Toronto, Department of Family and Community Medicine - Division of Emergency Medicine, Toronto, Canada

**Introduction**

A crisis currently facing the Emergency Department (ED) is overcrowding. It is not only a factor increasing the mortality of patients but also places a financial strain on the healthcare system. ED overcrowding is a complex issue with many factors at play, one being a patient’s waiting time in the ED. This research paper looked at the relationship between patient waiting time to their physician initial assessment (measured in minutes) and the time frame guidelines set by the Canadian Triage and Acuity Scale (CTAS).

**Methods**

This study was a retrospective analysis of data collected from Michael Garron Hospital’s ED over the year of 2015. 65,208 admissions were analysed. Using this data, the percentage of adherent and non-adherent patients in each triage group was compared, furthermore the day of the week that the non-adherent patients were seen, was recorded and graphed.

**Results**

Results revealed less than 50% of patients in triage groups 1 to 4 met the guidelines. Additionally, there was no distinct pattern between the day of the week and the number of patients exceeding the recommended CTAS guidelines however Monday does show the highest numbers of non-compliant patients.

**Discussion**

The results clearly showed poor adherence to guidelines. Moreover, despite results revealing the greatest discordance on Monday, no quantitative analysis done to show that this result was statistically significant. Lastly, the results from this study can perhaps encourage researchers to bring forward future ideas for patient waiting time improvement, as numerous consequences have been attributed to extended wait times.

## A15 John Bradley Clinical Research Program (JBCRP) 2017: A trans-atlantic community-based clinical research collaboration, ‘think globally, act locally’

### Yusuf Ahmed^1^, Heather Sampson^2^, Joyce Nyhof-Young^3^, Ruby Alvi^3^, Alice McGarvey^4^, Ann Hopkins^4^, Sarah Wright^2^, Cristina Leone^5^, John Abrahamson^2^

#### ^1^Faculty of Science, University of Western Ontario, London, Canada; ^2^University of Toronto/Michael Garron Hospital, Toronto, Canada; ^3^University of Toronto, Toronto, Canada; ^4^Royal College of Surgeons in Ireland, Dublin, Ireland; ^5^Michael Garron Hospital, Toronto, Canada

**Introduction**

International student integration potentially promotes research collaboration. The JBCRP arose from Michael Garron Hospital and Royal College of Surgeons in Ireland (RCSI) research grants to initiate and develop a mutually beneficial community-based international clinical research summer program for interdisciplinary students.

**Methods**

Students were selected from the University of Toronto Department of Family and Community Medicine, University of Ottawa, RCSI and Ontario’s Western University post graduate Clinical Research program. Students are required to describe, define and defend their supervised research projects at 7 mandatory weekly interactive clinical research sessions led by 21 senior clinical research and guest faculty and attend post session social events. Pre/post-session and post-program evaluations were conducted with students, faculty, and research supervisors.

**Results**

28/29 students from Canada, Ireland, Hong Kong and India completed the 2017 JBCRP. Deliverables include research project proposals and completions; conference abstract submissions, publications and workshops. Students completed YouTube One Minute Wonders outlining their projects and participated in a moderated poster presentation session; all completed a collaborative Knowledge Affirmation quiz. Students, faculty and supervisors continue to work collaboratively post-program. Faculty developed student excellence awards. Evaluation results demonstrated high JBCRP student satisfaction: recommended changes will be implemented.

**Discussion**

JBCRP combined learning and demonstrating core research competencies within clinical research projects manageable over eight weeks. Student evaluations recommend continuing the JBCRP; faculty and supervisors have committed to return. JBCRP produced local/international medical and inter-professional students knowledgeable and enthusiastic about applied clinical research and strong transatlantic education and research partnerships. Program sustainability is being investigated.

## A16 Use of appropriate signage and availability of personal protective equipment: A point prevalence study

### Brittany Telford^1^, Emma Moore^1^, Ellen Flynn^1^, Akshaya Ravi^1^, Raymond Healy^2^, Una Geary^2^

#### ^1^Trinity College Dublin, Dublin, Ireland; ^2^Quality and Safety Improvement Directorate, St James’ Hospital, Dublin, Ireland

**Introduction**

Hospital-acquired infections (HAIs) are a major cause of mortality and are a common adverse event associated with hospitalisation. The rate of isolation however, is currently unknown in Ireland. This point prevalence study aims to quantify the type of communicable diseases in isolation and evaluate the isolation precaution communication, availability of personal protective equipment (PPE) as well as other equipment necessary for maintaining isolation.

**Methods**

A standardised audit tool was developed in accordance with the National Standards for the Prevention and Control of Healthcare Associated Infections (May 2009). Data was collected by trained observers from March 14, 2017 to March 16, 2017, through observation of occupied isolation rooms in an academic teaching hospital in Dublin, Ireland. The data was subsequently used for additional analysis and discussion.

**Results**

Fourteen percent (125/869) of the total inpatient population was isolated. The most common isolation precaution was contact precautions (96.0%). Eighty-eight percent of known contact precautions were due to multi-drug resistant organisms (MDROs) and 13.3% of patients had more than one MDRO infection. 96% of patients requiring isolation were isolated, 92% of rooms had signage, 90.8% had appropriate signs and 93% of rooms had PPE available. Finally, thirty-one percent of rooms had patient-dedicated and single-use equipment.

**Discussion**

Overall, the majority of patients were isolated and communication of precautions was felt to be appropriate. However, the largest deficit was the availability of patient-dedicated and single-use equipment. This study highlights that evaluation of isolation precautions is essential in determining compliance with hospital guidelines and is recommended for standard practice.

## A17 The effect of TNF-alpha in injured mice serum on vascular endothelial growth factor level of mesenchymal stem cells

### Fitri Hayuningtyas^1,2^, Anggun Prasetyowati^1,2^, Evan Tantono^1,2^, Fikri Faisal Haq^1,2^, Farah Fauziah^1^, Sigit Harya Hutama^1,2^, Nugraha Wirawan^1,2^, Ika Rosdiana^3^, Agung Putra^2^

#### ^1^Medical Faculty, Sultan Agung Islamic University, Semarang, Indonesia; ^2^Stem Cell and Cancer Research, Sultan Agung Islamic University, Semarang, Indonesia; ^3^Medical Rehabilition, Medical Faculty of Sultan Agung Islamic University, Semarang, Indonesia

##### **Correspondence:** Agung Putra

**Introduction**

Mesenchymal Stem Cells (MSCs) is widely used to treat various diseases. The success of MSCs therapy is achieved when MSCs is activated by mediators such as TNF-alpha (TNF-α). The activated MSCs will secrete Vascular Endothelial Growth Factor (VEGF). The aim of this study is to analyze VEGF levels of MSCs that activated by TNF- α in injured mice serum.

**Methods**

Injured mice serum was analyzed to observe the level of TNF-α. The experimental groups (serum concentrations of 25%, 12.5%, and 6.25%) and control were incubated to MSCs for 48 hours. VEGF level of each group was measured by Enzyme Linked Immunosorbent Assay (ELISA) with wavelength of 450 nm.

**Results**

A significant increase in TNF-α level was found in injured mice serum. VEGF level significantly increased at 6.25% serum concentration with VEGF level 30.86 pg/ml (p=0.003). VEGF level significantly decreased to 27.77 pg/ml (p=0.000), 27.2 pg/ml (p=0.004) in 12.5% and 25% serum concentrations.

**Discussion**

Lowest concentration of TNF-α in injured mice serum may increase VEGF level through nFKB pathway. The higher concentration of TNF-α in injured mice serum is predicted to induce apoptosis by activation of TNF Receptor 1 (TNFR1) as death receptor.

## A18 Decreased serum and cardiac thyroid hormones in an animal model of HFpEF

### Catarina Vale, Adelino Leite-Moreira, João Neves, Inês Falcão-Pires, André Lourenço

#### Department of Surgery and Physiology, Faculty of Medicine, University of Porto, Porto, Portugal

**Introduction**

Heart failure with preserved ejection fraction (HFpEF) is a frequent disease and its hallmark is diastolic dysfunction. Diminished thyroid hormones are known to promote diastolic dysfunction. The aim of this study was to characterize the thyroid function in an animal model of HFpEF.

**Methods:**

Thyroid hormones T3 and T4 were quantified in the serum, the left ventricle and the visceral adipose tissue of ZSF1 Obese rats (HFpEF) and ZSF1 Lean rats (controls) by radioimmunoassay. Serum TSH was also quantified by ELISA.

**Results**

Both serum (Lean T3 = 35,85 ± 9,39 ng/dL and T4 = 3,49 ± 1,35 μg/dL; Obese T3 = 5,96 ± 4,65 ng/dL and T4 = 1,51 ± 0,64 μg/dL) and left ventricle levels of thyroid hormones (Lean T3 = 10,51 ± 7,91 ng/g and T4 = 2,02 ± 0,60 ng/g; Obese T3 = 3,87 ± 0,85 ng/g and T4 = 0,99 ± 0,43 ng/g) were significantly decreased in ZSF1 Obese rats. The visceral adipose tissue levels of T3 and T4 (Lean T3 = 1,52 ± 0,88 ng/g and T4 = 2,77 ± 1,39 ng/g; Obese T3 = 0,98 ± 0,69 ng/g T4 = 2,20 ± 0,76 ng/g) and the serum TSH levels (Lean 0,65 ± 0,38 ng/mL; Obese 0,79 ± 0,57 ng/mL) were not significantly different between the groups.

**Discussion**

These results are consistent with euthyroid sick syndrome in ZSF1 Obese rats (lower thyroid hormones and normal TSH levels). The low levels of thyroid hormones may contribute to diastolic dysfunction and, therefore, exacerbate HFpEF.

## A19 Investigating the levels of IL-17 in the lungs of patients with cystic fibrosis

### Ishapreet Kaur^1^, Emer P Reeves^2^, Michelle White^2^

#### ^1^Faculty of Medicine, Royal College of Surgeons in Ireland, Dublin, Ireland; ^2^Irish Centre for Genetic Lung Diseases, Medicine, Beaumont Hospital, Dublin, Ireland

**Introduction**

Cystic fibrosis (CF) is a genetic condition characterised by dysregulated neutrophil inflammation within the lung. Our group demonstrated that CF neutrophil membrane metalloprotease ADAM-17 cleaves IL-6 receptor to a soluble form, when complexed with IL-6 induces the production of Th17 cells. We hypothesized that high levels of IL-17A are found within the airways of patients with CF (PWCF). The objective of the study was to investigate the levels of IL-17A/F in the bronchoalveolar lavage fluid (BALF) isolated from PWCF.

**Methods**

Ethical approval was obtained from Beaumont Hospital (Ref Number 14/98) for recruitment of patients with CF and healthy individuals (HC) for BALF and plasma (n=12). ELISA quantified IL-17. Active neutrophil elastase (NE) was quantified using FRET assay. Cleavage experiments of IL-17 were carried out using NE, HC and CF BALF, at different time points. Statistical analysis was determined using student t-test and one-way ANOVA followed by Tukey’s post hoc test.

**Results**

IL-17 levels were not elevated in plasma isolated from PWCF (p=ns). IL-17 was reduced in BALF of PWCF (p=0.002). Recombinant IL-17 incubated with pooled CF BALF had significantly reduced IL-17 levels compared to HC BALF (p<0.01). CF BALF had significantly more active NE when compared to HC BALF (p=0.016) which we demonstrated to cleave IL-17 (p<0.001).

**Discussion**

This study demonstrated that IL-17 levels are decreased in the lungs of PWCF. Our results revealed that active NE cleaves IL-17. IL-17 plays a protective role in the lungs. Therefore, its cleavage in the lung of PWCF could result in sustained and chronic inflammation.

## A20 Are adverse psychological outcomes more likely in patients with cardiac implantable electronic devices (CIED)?

### Hamsa Vahini Mahadevan^1^, Martin Stiles^2^

#### ^1^Department of Cardiology, University College Dublin, Dublin, Ireland; ^2^Department of Cardiology, Waikato Hospital, Waikato, New Zealand

**Introduction**

There is considerable uncertainty about the impact of Cardiac Implantable Electronic Devices (CIED) on the overall psychological wellbeing of patients. Clinical depression and anxiety are major adverse psychological outcomes and can add additional impairment to the quality of life for CIED patients. The aim of this research was to (a) investigate the quality of life of patients with CIED and (b) assess the treatment satisfaction of patients.

**Methods**

100 CIED patients (24 ICD and 76 pacemaker) were questioned regarding their device and treatment satisfaction. Their levels of depression and anxiety were quantified using the Hospital Anxiety and Depression Scale (HADS).

**Results**

The prevalence of clinical depression and anxiety based on the scores were 7% and 15%, respectively. The mean anxiety and depression scores were 2.78 and 3.29, respectively; which reflected levels similar to the general population. These results are consistent with the patient’s satisfaction from treatment; 60% of the patients felt better after the device implantation, while 38% felt the same. 70% of the patients took less than a month to adapt to their devices. 84% of the patients felt it was worthwhile having the device implanted.

**Discussion**

These results demonstrate that these patients have insignificant levels of depression or anxiety and are highly satisfied with the treatment. The small sample size and lack of correlation with other clinical markers (e.g. underlying cardiac condition, co-morbidities and presence or absence of device therapy or malfunction) is a significant limitation. Recruiting continues to hopefully address this issue.

## A21 Readouts of aerobic glycolysis in cystic fibrosis correlate with CF-ABLE score

### Johanna Pinto Lee, Emer Reeves, Oliver McElvaney, Noel G McElvaney

#### Irish Centre for Genetic Lung Diseases, Royal College of Surgeons in Ireland, Beaumont Hospital, Dublin, Ireland

**Introduction**

Cystic fibrosis (CF) is a genetic disorder characterized by a mutation in the cystic fibrosis transmembrane conductance regulator (CFTR) gene. Cycles of inflammation and infection are hallmarks of CF. The CF-ABLE score is a clinical measure of disease severity in CF. As a key pro-inflammatory cytokine, IL-1β is mediator of inflammation. IL-10 is an anti-inflammatory cytokine which inhibits pro-inflammatory cytokines. In aerobic glycolysis, dimerization of pyruvate kinase M2 allows translocation to the nucleus and stabilization HIF1a. This culminates in upregulation of pro-inflammatory cytokines (IL-1β) and downregulation of anti-inflammatory cytokines (IL-10). These experiments hypothesized that excessive inflammation observed in CF can be in part attributed to a shift towards IL-1β and away from IL-10, and that this shift can be correlated with CF-ABLE score.

**Methods**

CF-ABLE scores were calculated using patient records. BALF was collected from patients with CF, healthy controls, primary ciliary dyskinesia patients and post-transplant CF patients. Plasma was collected from patients with CF and healthy controls. IL-1β and IL-10 levels were quantified by ELISA.

**Results**

IL-1β levels in CF BALF were increased compared to HC, PCD and post-transplant CF BALF, with IL-1β levels in CF BALF post lung transplantation comparable to HC (P=0.0002). The plasma IL-1β:IL-10 in CF is also increased (P=0.008). IL-1β levels in BALF correlated strongly with CF-ABLE score (R^2^ = 0.9562).

**Discussion**

IL-1β to CF-ABLE score correlation provides a molecular measure for disease severity, complementing the CF-ABLE score’s clinical applications. IL-1β levels link molecular mechanisms of aerobic glycolysis to clinical applications of the CF-ABLE score.

## A22 Comparison between clinical diagnosis of catheter-associated urinary tract infections and National Healthcare Safety Network’s criteria

### Jonathan Cheung^1^, Lin Li^2^, Leyland Chuang^2^

#### ^1^School of Medicine, Royal College of Surgeons in Ireland, Dublin, Ireland; ^2^Ng Teng Fong, Department of Medicine, General Hospital, Singapore, Singapore

**Introduction**

Urinary tract infections (UTIs) are the commonest cause of nosocomial infections, of which a quarter are catheter-associated UTIs (CAUTIs). An estimate of twenty five percent of hospitalized patients have urinary catheters. Definitions of CAUTIs remain controversial, but the National Healthcare Safety Network (NHSN) has defined a criterion. This study aims to compare the clinical diagnosis of CAUTIs against the NHSN criterion, at a general hospital in Singapore.

**Methods**

A retrospective audit was performed on 1436 electronic medical records of urinary catheters placed between February 1st and April 30th, 2016. Records were assessed for a clinical diagnosis CAUTI, and also against the NHSN CAUTI criteria. Data obtained included: patient admission and discharge dates, catheter insertion and removal dates, urine cultures, documented clinical diagnosis of UTI, UTI symptoms, and the date of event.

**Results**

Of 1436 patients with urinary catheters, 33 cases (2.30%) had a clinical CAUTI diagnosis, and 34 (2.37%) cases met NHSN CAUTI criteria. However, only 5 cases of clinical CAUTI fulfilled NHSN criteria, corresponding to a positive predictive value of 15.15% (95% confidence interval, 6.84 to 30.28%). 15 cases (45.5%) of clinical CAUTI had no symptoms and 10 cases (69.7%) did not have positive urine cultures.

**Discussion**

This audit highlighted a discordance in clinical diagnosis of CAUTI compared to NHSN’s criteria of CAUTI. This suggests that clinicians are misdiagnosing CAUTIs, which may lead to both insufficient, and also unnecessary antimicrobial prescriptions.

## A23 INR targets for mechanical valves: A systematic review and meta-analysis

### Saurabh Gupta^1,2^, Emilie Belley-Cote^2,3,4^, Anisha Sarkaria^6^, Arjun Pandey^8,11^, Jessica Spence^4,9^, Graham McClure^4,10^, Puru Panchal^11^, Iqbal Jaffer^3,8^, Kevin An^10^, John Eikelboom^5,6^, Richard Whitlock^3,4,6^

#### ^1^McMaster University/Population Health Research Institute/Hamilton General Hospital, Cardiac Surgery, Hamilton, ON, Canada; ^2^Department of Health Research Methods, Evidence, & Impact, McMaster University, Hamilton, ON, Canada; ^3^Department of Medicine, McMaster University, Hamilton, ON, Canada; ^4^Population Health Research Institute, Hamilton, ON, Canada; ^5^Queen’s University Medical School, Kingston, ON, Canada; ^6^Thrombosis & Atherosclerosis Research Institute, Hamilton, ON, Canada; ^7^Department of Anesthesia, McMaster University, Hamilton, ON, Canada; ^8^Michael DeGroote School of Medicine, McMaster University, Hamilton, ON, Canada; ^9^Faculty of Health Sciences, McMaster University, Hamilton, ON, Canada

**Introduction**

Guidelines recommend higher INR targets for patients with mechanical valves who are believed to be at higher risk for thromboembolism. Higher INR targets are associated with increased bleeding risk. We performed a systematic review and meta-analysis assessing effects of lower and higher INR targets on thromboembolism and bleeding risk in patients with mechanical heart valves.

**Methods**

We searched Cochrane CENTRAL, MEDLINE and EMBASE for randomized controlled trials (RCTs) and observational studies evaluating lower versus higher INR targets for adults with bileaflet mechanical valves. We performed title and abstract screening, full-text review, risk of bias evaluation, and data collection independently and in duplicate. We evaluated overall quality of evidence with the GRADE framework, and pooled data using a random effects model.

**Results**

We identified 6 RCTs (n = 5497) and 2 observational studies (n = 1195). In RCTs, lower INR targets were associated with significantly less bleeding – 22% versus 40% – (RR 0.54, 95%CI 0.31, 0.93, p=0.03, very low quality). There was no difference in thromboembolism – 2% in both groups (RR 1.28, 95%CI 0.88, 1.85, p=0.20, very low quality) or mortality – 5.5% with lower INR targets versus 8.5% (RR 1.00, 95%CI 0.82, 1.21, p=0.47, moderate quality). Observational studies showed no significant differences in bleeding or thromboembolism, whereas lower INR targets were associated with lower mortality (RR 0.56, 95%CI 0.49, 0.63, p<0.00001).

**Discussion**

In patients with bi-leaflet mechanical valves, lower INR targets appear to be associated with lower bleeding risk when compared to higher INR targets without compromising efficacy.

**Acknowledgements**

This poster was presented by Sarah MacIsaac on behalf of Dr. Richard Whitlock’s MiNION research group.

## A24 Promising novel antimicrobials: Star-shaped nano-engineered antimicrobial peptide polymers (SNAPPs) against multi-drug resistant pathogens

### Richard Tan^1^, Deirdre Fitzgerald-Hughes^1^, Andreas Heise^2^, Robert Murphy^2^

#### ^1^Department of Clinical Microbiology, Royal College of Surgeons in Ireland, Beaumont Hospital, Dublin, Ireland; ^2^Department of Pharmaceutical and Medicinal Chemistry, Royal College of Surgeons in Ireland, Dublin, Ireland

**Introduction**

Treatment of infection is increasing challenging due to rising antibiotic resistance and new agents are needed. Star-shaped Nano-engineered Antimicrobial Polypeptide Polymers (SNAPPs) have potential for development as alternative antimicrobials as their bacterial targets are distinct from those of conventional antibiotics.

**Methods**

The susceptibility of clinical isolates of pathogenic bacteria to the 64-arm SNAPP, G5(S64)–PLL5 was investigated. Minimum inhibitory concentrations (MIC) and bactericidal activity of G5(S64)–PLL5 was determined. The effect of human plasma on bactericidal activity was also evaluated.

**Results**

MIC values of 12-50μm were found against Gram-negative bacteria (multi-drug resistant (MDR) Klebsiella pneumoniae and Escherichia coli). Lower MICs (< 0.195 μm) were found for the Gram-positive bacteria tested, a linezolid-resistant, vancomycin resistant Enterococcus faecium (VRE). Potent bactericidal activity was also found (90% killing at 25, 2.5, 10 μm for K. pneumoniae, E. coli, MDR E. coli respectively, and at 50nM for VRE, n=3). The addition of human plasma (20% v/v) to assays, reduced the bactericidal activity of G5(S64)–PLL5 against VRE but bactericidal activity was maintained for all other isolates in the presence of plasma.

**Discussion**

This study demonstrated potent antibacterial activity of G5(S64)–PLL5 against all bacteria tested including isolates resistant to multiple antibiotics. Linezolid-resistant VRE are an emerging threat for which treatment options are increasingly limited, therefore the finding of SNAPP activity against VRE in the low nM range is encouraging. Further investigations, particularly in in-vivo relevant conditions will be important in the further development of SNAPPs as novel antimicrobial agents.

## A25 Comprehensive strategies in the treatment of chronic prostatitis

### Burra Mithilesh Bharadwaj^1^, Reznichenko Yurievna^2^, Vinisha Tekwani^3^, Varahabhatla Vamsi^3^

#### ^1^General Medicine, Faculty of Medicine, Zaporozhye, Ukraine; ^2^Department of Dermatology and Venerology, Zaporozhye, Ukraine; ^3^General Medicine, Zaporozhye, Ukraine

**Introduction**

Prostatitis patients are increasingly exploring complementary alternative medicine due to the risk of adverse events from standard treatment. The project aims to determine effectiveness of phytopreparations for chronic prostatitis and age-related changes of prostate gland.

**Methods**

67 male patients were examined. Cucumber extract was given in variable doses for definitive manifestations: 2 tablets TID for 3 months for chronic pelvic pain; 3 tablets TID for 1 month followed by 2 tablets TID for 2 months for prostatitis with asymptomatic course; 4 tablets TID for 2 weeks by decreasing to 3 tablets TID for 3 months for benign prostatic hyperplasia and 2 tablets TID for 2 months for age-related changes in prostate in men older than 35 years.

**Results**

The use of cucumber extract improved the effectiveness of basic therapy by 78,6%. Effectiveness was noted in 10 days of treatment. Pain syndrome reduced by 25.0% in 5 days and to 30.9% in 10 days, when compared to patients receiving standard treatment. The incidence of dysuric phenomena deducted by 29.6% in 5 days, whereas in 10 days decreased by 42.6%. The number of leukocytes in the secretions of the prostate gland was 23.4% lower in patients with chronic bacterial prostatitis, who used Prostamed, than in control group. Assessment of quality of life after 1 month was 18.8% higher in patients, who used Prostamed, compared with the control group.

**Discussion**

Cucumber extract prevented the occurrence of benign hyperplasia, prostatitis and age-related changes in the prostate gland. It is advisable to introduce cucumber derivatives in clinical practice.

## A26 Activation of hypothalamic μ-opioid receptors induces tumour suppression

### Shivran Singh^1^, Yusuke Hamada^2^, Minoru Narita^2^

#### ^1^Faculty of Medicine, Royal College of Surgeons in Ireland, Dublin, Ireland; ^2^Department of Pharmacology, Hoshi University, Ebara, Shinagawa-Ku, Tokyo, Japan

**Introduction**

Opioids mainly act on the μ-opioid receptor (MORs), localized in the brain, producing their analgesic effects. Recent studies have identified the involvement of peripheral opioid receptors in altering tumour cell proliferation and cancer metastasis. However, little is known about the possible involvement of central MORs in tumour growth. Aim: To investigate the role of opioids in tumour progression and alteration of the tumour microenvironment via activation of the central μ-opioid receptor, in the hypothalamus.

**Methods**

Our lab generated transgenic mice with the designer receptor human M3 muscarinic receptor (hM3Dq) via the Designer Receptors Exclusively Activated by Designer Drugs (DREADD) system. Lewis Lung Carcinoma (LLC) cells were implanted into the right lower limb of these hM3Dq transgenic mice and allowed to propagate before commencing a 2-week course of systemic injection with TRV130. Tumour volume was then be measured and assessed by comparison to the control group.

**Results**

Tumour volume was observed to be reduced in hM3Dq transgenic mice bearing LLC when treated with the G-protein-biased ligand, TRV130. This is because TRV130 is believed to induce tumour suppression, similar to morphine via activation of central μ-opioid receptors in the hypothalamus.

**Discussion**

The present findings suggest that the opioid, TRV130, may suppress tumour growth via activation of the hypothalamic Arcuate Nucleus-Hypothalamic Pro-opiomelanocortin (ARC-POMC) neurones and inhibition of the Paraventricular Nucleus-Corticotropin-releasing hormone (PVN-CRH) neurones, by binding to central μ-opioid receptors in the hypothalamus. This may then alter the HPA axis-dependent stress response to cancer by inhibiting the release of CRH and thus altering the surrounding tumour microenvironment.

## A27 Comparison of pain limit of patients with tension-type headache with pericranial sensitivity and patients with sciatica measured with digital algometer

### Filip Kaianić, Sandro Kalember, Aleksandar Kopitović, Svetlana Simić

#### Department of Neurology, Faculty of Medicine, University of Novi Sad, Novi Sad, Serbia

**Introduction**

Tension-type headache and sciatica are one of the most common causes of work disability, which is why they have a very important socio-economic impact.

**Methods**

A prospective study was conducted which included 59 patients. As a source of medical information about patients we used medical documentation with clinical information. With pain detect, neuropathic pain was confirmed and pain limit was measured with digital algometer.

**Results**

Pain limit of head points in patients with headache is 179 kPa/cm2, in patients with sciatica is 285 kPa/cm2 and between them there is no statistically significant difference. In healthy patients, this limit is 455 kPa/cm2 and there is statistically significant difference between them and the other groups. Pain limit of lumbar points in patients with sciatica is 424 kPa/cm2, in patients with headache is 477 kPa/cm2 and between them there is no statistically significant difference. In healthy patients, this limit is 803 kPa/cm2 and there is statistically significant difference between them and the other groups. Pain limit of leg points in patients with sciatica is 409 kPa/cm2, in patients with headache is 511 kPa/cm2 and between them there is no statistically significant difference. In healthy patients this limit is 672 kPa/cm2 and there is statistically significant difference between them and the other groups.

**Discussion**

The aim was to determine and compare pain limit of patients with tension-type headache, patients with sciatica and healthy volunteers. Patients with chronic pain syndrome have diffuse lowered pain limit in whole body.

## A28 Exploring caregiver knowledge and attitudes towards deprescribing antipsychotics in dementia

### Sri Vatturi^1,2^, Christopher Smith^2,3^

#### ^1^Faculty of Medicine, Royal College of Surgeons in Ireland, Dublin, Ireland; ^2^Michael Garron Hospital, Toronto, Canada; ^3^Department of Medicine, University of Toronto, Toronto, Canada

**Introduction**

Antipsychotics are poorly efficacious and not approved for treating behaviors in dementia; however the off-label use for this indication has increased in the past twenty years. Caregivers often believe antipsychotics are effective and safe. Exploring their knowledge and attitudes is an important step in designing a successful antipsychotic deprescribing program.

**Methods**

All patients admitted to a medical service at the Michael Garron Hospital from July 4 to August 11, 2017 were screened and those 65 years old and above with a diagnosis of dementia were included in the study. A questionnaire was created to assess the caregiver’s knowledge and attitudes towards pharmacologic and non-pharmacologic therapies for dementia related behaviors.

**Results**

47% of admitted dementia patients were on antipsychotics. 79% of caregivers knew why antipsychotics were prescribed but only 23% were aware of the side effects. Caregiver openness to taking patient off antipsychotics was rated as only 4.8/10. 50% of caregivers had heard of non-pharmacologic treatments for behaviors, and 13% had tried them. The most commonly known and used were music therapy and cognitive rehabilitation. Caregivers rated knowledge of both antipsychotics and non-pharmacologic therapies low, at 1.9/5 and 1.7/5 respectively. The average rating for wanting to know more about non-pharmacologic therapies was 8.3/10.

**Discussion**

There are knowledge gaps, which may be contributing to the caregiver’s resistance to stopping antipsychotics. Adding an education component to deprescribing initiatives is likely to be helpful.

## A29 Improving access to care for emergency department patients presenting with dizziness

### Jeffrey Lam^1^, Bradley Hubbard^2,3^, Paul Hanam^4^, Sarah Chiodo^5^, Cameron Neeson^6^, Albino Chiodo^2,3^

#### ^1^Royal College of Surgeons in Ireland, Dublin, Ireland; ^2^Department of Otolaryngology-Head & Neck Surgery, Michael Garron Hospital, Toronto, Canada; ^3^Department of Medicine, University of Toronto, Toronto, Canada; ^4^Department of Emergency Medicine, Michael Garron Hospital, Toronto, Canada; ^5^Michael Garron Hospital, Toronto, Canada; ^6^Cornell University, Ithaca, New York

**Introduction**

Overcrowding continues to be a significant problem in emergency departments (ED) worldwide. Acute dizziness is one of the most common presenting complaints of ED patients and can be caused by a variety of pathologies, each with distinct associated symptoms. Referral of care varies according to the underlying cause and incorrect referrals are costly in time and resources. This quality improvement project aims to streamline Michael-Garron Hospital (MGH) ED patient access to care by developing the first questionnaire for dizziness patients at MGH.

**Methods**

Ethics approval was obtained as per MGH protocol. A questionnaire was made based upon existing questionnaires from other healthcare facilities—collecting data on demographics, associated symptoms, and past-medical history. Dizziness patients completed the questionnaire as they waited in the ED Green Zone. Collected data is compared with the final diagnosis to determine questionnaire effectiveness in predicting accurate referral of care. Statistical analysis is also performed to observe potential correlations and pathology prevalence based on demographics.

**Results**

Data on presenting-complaint, associated symptoms, medication, medical history, and family history were collected from 71 dizziness patients via a questionnaire designed for this purpose. Potential referral from the questionnaire was compared with the real-life diagnosis and referral. Statistical analysis is currently ongoing, expected for completion in November 2017.

**Discussion**

This QI project aims to develop a questionnaire for MGH ED dizziness patients to expedite access to care and reduce percentage of incorrect referrals. Future studies may focus on other common presenting complaints, migration to electronic questionnaires, and standardized intake forms across local hospitals.

## A30 Knowledge, attitude and preventive practices towards cervical cancer among female undergraduates in a tertiary institution in Ogun state, Nigeria

### Gloria Ben-Okoh^1^, Olanrewaju Onigbogi^2^, Folu Olatona^2^

#### ^1^Department of Medicine & Surgery, College of Medicine of the University of Lagos, Nigeria; ^2^Department of Community Health and Primary Care, College of Medicine of the University of Lagos, Nigeria

**Introduction**

Cervical cancer remains a major health problem in developing countries. In Nigeria, it is the second leading cause of female cancer and female cancer deaths; however, research has shown that it is preventable. This study assessed the knowledge of cervical cancer, Human Papillomavirus (HPV) vaccination and cervical cancer screening. Respondents’ attitudes and preventive practices were also assessed.

**Methods**

This was a descriptive cross-sectional study that used multistage sampling technique to select 280 female undergraduates in Federal University of Agriculture, Abeokuta, Ogun state. Data was collected using a pretested, self-administered questionnaire and analysed using Epi Info.

**Results**

The mean age of the respondents was 20±2 years. Most (77.86%) were aware of cervical cancer, however only 27.50% and 32.14% were aware of HPV vaccination and cervical cancer screening respectively. Good knowledge of cervical cancer, HPV vaccination and screening was demonstrated by 17.43%, 19.48% and 42.2% of respondents respectively. Majority (90.36%) had a positive attitude towards cervical cancer and its preventive measures. Only nine of the 280 respondents had received HPV vaccination and four had any screening for cervical cancer done. Most of the respondents were willing to be vaccinated (69.64%) and participate in a screening program (73.57%) if it was free.

**Discussion**

Knowledge of risk factors, symptoms and preventive measures were poor. Knowledge of preventive measures did not translate to the uptake of vaccination and screening in this study. This information is important for the development and implementation of policies and programs to improve knowledge and preventive practices towards cervical cancer.

## A31 The importance of sexual education of medical students

### Stavroula Papaeleftheriou, Melindi Brink, Martha Gismondi, Marc A. Heller, Olympia E. Chatzigianni, Estefania H. Cases

#### Department of Public Health, University of Medicine and Pharmacy “Gr. T. Popa” Iasi, Iasi, Romania

**Introduction**

In recent times it seems that there is a decrease of stigmata regarding the discussion of sexual attitude, preference, functions and dysfunctions. On the contrary, medical graduates appear to be insufficiently trained during university in topics such as Sexual Transmitted Infections (STIs), AIDS or HIV, reproductive or pregnancy-related pathologies, sexual violence, sexual preference and psychopathologies related to sexual attitudes.

**Methods**

A survey was conducted among medical students over the past few months in an effort to observe whether undergraduate medical students are receiving proper education in order to face and treat sexual health issues. This survey was published in various groups of certain social websites and it included 22 questions, of which 13 questions could be answered by the participants with a satisfaction scale. This Survey was conducted anonymously.

**Results**

The survey was answered by 272 medical students. Of these, 95.2% agreed with the importance of medical professionals taking a sexual history. However, on the question “I feel comfortable discussing sexual health problems with patients of opposite sex” 29.4% answered with neither agree or disagree and 26.7% answered that they find taking a sexual history hard. Unfortunately, 46% of the participants found that they were not properly trained during their medical school.

**Discussion**

Medical schools worldwide seem to lack sexual health training hours within their syllabus. This limits future doctors in providing good medical care in sexual health matters. Additionally, this results in a lack of confidence, but also a lack of interest, since it was not sufficiently covered in their education.

## A32 Quantitative sensory assessment using current perception threshold with a peripheral nerve stimulator

### Jeremy H Tsui^1^, Gareth Corry^2^

#### ^1^School of Medicine, Royal College of Surgeons in Ireland, Dublin, Ireland; ^2^Department of Anesthesiology and Pain Medicine, University of Alberta, Edmonton, Canada

**Introduction**

A quantitative method for assessing peripheral nerve blocks would provide a graded trend and minimize the subjectivity inherent to traditional “all or none” methods (e.g., ability to detect cold stimulus) that rely on patient feedback. Recently, we demonstrated that the quantitative measurement for current perception threshold (CPT) could be achieved with a peripheral nerve stimulator. In this study, we aligned the clinical results of an ice test (i.e., an “all or none” method) and CPT measurements made with a similar peripheral nerve stimulator.

**Methods**

In this pilot study, we recruited 29 patients who required a supraclavicular brachial plexus nerve block for hand surgery. Using a peripheral nerve stimulator, we obtained CPT measurements for both the median (second digit) and ulnar (fifth digit) nerve distributions before the block was given and then every five minutes thereafter until the patient could not feel a cold sensation on both digits when ice was applied. Receiver-operating characteristics (ROC) curves and Youden indices were calculated for each device to evaluate diagnostic effectiveness.

**Results**

The area under the ROC curve was 0.887 (95% CI 0.835-0.938). Calculation of the Youden index showed that the peak J value (0.652) occurred at approximately double the baseline CPT value (1.95).

**Discussion**

Our results suggest that, when post-block CPT values are twice the level of pre-block values, the block may be deemed sufficient to proceed with surgery. Further studies are required to confirm if the nerve stimulator settings used in this study are applicable to other blocks.

## A33 Usage of prophylactic anti-emetics during caesarean section under regional anaesthesia: A prospective clinical audit

### Feargal Donaghy^1^, Caitriona Murphy^2^

#### ^1^Department of Research, Royal College of Surgeons in Ireland, Dublin, Ireland; ^2^Anaesthetics, Rotunda Maternity Hospital, Dublin, Ireland

**Introduction**

Postoperative nausea and vomiting (PONV) is a recognised side effect of intrathecal/epidural opioids for lower segment caesarean sections (LSCS) under regional anaesthesia. NICE guidelines 2011 recommend offering prophylactic antiemetics post chord clamping in this patient population. This audit reviews intraoperative antiemetic administration, estimates antiemetic impact on PONV incidence and makes recommendations in line with best practice.

**Methods**

A prospective chart audit was performed in the Rotunda Maternity Hospital, Dublin. Patients 24 hours post LSCS who received intrathecal/epidural opioids were included. Patients who received general anaesthesia (GA) or had foetal/maternal adverse outcomes were excluded. Approval was obtained by the Rotunda Audit Committee.

**Results**

Of the 332 included patients, 43% received intraoperative antiemetic medication and 37.65% reported PONV. Compared with patients for whom prophylactic antiemetics were omitted, patients receiving at least one intraoperative antiemetic had a 15.26% absolute risk reduction of PONV (44.04% vs 28.78% respectively), and a 34.66% relative risk reduction for PONV (RR 0.65, p= 0.0064, 95% CI 0.48-0.89, NNT= 6.5), in addition to a 11.2% absolute risk reduction (24.87% vs 13.67% respectively) and a 45.04% relative risk reduction for rescue antiemetics (RR 0.56, p=0.0154, 95% CI 0.34-0.89, NNT=8.9). A higher incidence of PONV in patients with a previous history of PONV post GA and post LSCS (25.24% and 23.75% respectively) compared to those with no prior history, was also observed.

**Discussion**

This audit supports routine prophylactic antiemetics to reduce PONV incidence and rescue antiemetic requirement. Prior history of PONV post GA or LSCS represents a particular risk warranting special consideration.

## A34 Analysis of the results of surgical methods for treating of primary tumors and tumor-like lesions of bone in children

### Elyarbek Tashmetov, Maksim Em, Said Kuziev, Aleksandr Shlegel, Sergei Kasyanov

#### General Medicine, Karaganda State Medical University, Karaganda, Kazakhstan

**Introduction**

Although primary bone tumors are relatively uncommon, they represent the 3rd leading tumor type in younger populations with important diagnostic and therapeutic implications. Study was conducted to evaluate the effectiveness of surgical treatment with primary tumors and tumor-like lesions of bone in children.

**Methods**

A total of 35 (26 males, 9 females) patients with a mean of 12.7 (aged 7 – 17) years. Exostosis was the commonest tumor accounting for 12 cases followed by osteoma – 7, fibrous dysplasia – 6, osteoid osteoma – 4, osteochondroma – 2, aneurismal bone cyst – 2, osteoblastoma – 1, non-ossified fibromas – 1. Bone tumors 25 (71.4%) occurred in the lower extremities, while 10 (28.6%) were in the upper extremities. A surgical procedure was performed in 35 patients. Twelve patients (34.3%) were required for repairing and reconstructing bone defect after resection. Depending on size of cavity, they were reconstructed in 8 cases by autograft, in 2 by frozen allograft, in 2 were combined. The results were evaluated by clinical and X-ray data. The follow up period varied 6 – 12 months.

**Results**

Good results were obtained in 34 (97.1%) patients. One patient with an aneurysmal cyst had a relapse of the disease. In 2 patients with fibrous dysplasia were found new foci after 6 months. The patients were re-operated.

**Discussion**

Priority in the treatment of benign tumors and tumor-like lesions of the bone in children is the performance of organ preserving surgery. The differential approach in choosing the variants of osteoplasty and their combination allow to achieve good results in all patients of this group.

## A35 The impact of arm dominance on the clinical and surgical outcomes in patients with thoracic outlet syndrome

### Amenah Dhannoon^1^, Husain Alshaikh^2^, Ying Wei Lum^2^

#### ^1^School of Medicine, Royal College of Surgeons in Ireland, Dublin, Ireland; ^2^Division of Vascular Surgery and Endovascular Therapy, Johns Hopkins Hospital, Baltimore, Maryland, USA

**Introduction**

Thoracic Outlet Syndrome (TOS) refers to the compression of the brachial plexus, subclavian artery and vein during their passage between the thorax and the upper extremity resulting in upper extremity impairment. There are three subtypes of TOS: Neurogenic TOS (NTOS), Venous TOS (VTOS) and Arterial TOS (ATOS). Our study is the first study to examine the impact of arm dominance on the clinical and surgical outcomes of patients treated for TOS.

**Methods**

Demographics, hand dominance, TOS type, duration of symptoms and surgical outcomes were retrospectively reviewed for 863 patients who underwent surgical decompression at the Johns Hopkins Medical Institution in the last 14 years.

**Results**

A total of 275 (42%) patients had TOS in the non-dominant arm and 380 (58%) patients had TOS in the dominant arm. There was no difference between the two groups in baseline characteristics. Although proportion of patients with NTOS tended to be higher on the non-dominant arm (61.5% vs 56.3%) and VTOS tended to be higher on the dominant arm (37.6% vs 30.5%), there was no statistically significant difference in overall incidence of TOS subtypes. Median duration of symptoms before referral was slightly higher for the non-dominant (18 vs 12 months). Successful surgical outcomes were more likely to occur in the dominant group (90.6% vs 84.4%, p=0.032).

**Discussion**

More patients had TOS on the dominant arm than the non-dominant as a result of the body’s physiological adaptations. Also, positive surgical outcomes were higher when TOS was on the dominant arm, which can be explained by the earlier presentation.

## A36 Investigation of the recovery and stability of PLGA-PEG nanoparticles for drug delivery in inflammatory bowel disease

### Fayez Aldhufairi^1^, Jacqueline Daly^1^, Zeibun Ramtoola^2^, Lauren Mohan^1^

#### ^1^Medicine, Royal College of Surgeons in Ireland, Dublin, Ireland; ^2^Pharmacy, Royal College of Surgeons in Ireland, Dublin, Ireland

**Introduction**

The term inflammatory bowel disease (IBD) is used to describe Crohn’s disease (CD) and ulcerative colitis (UC). While there are a wide range of therapeutic options available for the treatment of IBD, there is an unmet clinical need for more targeted and effective therapies. In recent years, research has been focusing on the development of nanoparticle (NP)-based agents to provide targeted drug delivery. NPs are defined as structures which range between 1-1000 nm in size. Currently the applications of NPs in medicine include drug delivery and both in vitro and in vivo diagnostics. NPs demonstrate enhanced penetration of inflamed intestinal tissue over healthy tissue and therefore represent an exciting and novel drug delivery strategy for IBD treatment. Nevertheless, NP instability is a major obstacle which can limit their uses.

**Methods**

Many pharmaceutical products use freeze-drying process to improve upon formulation stability. There are only few studies that examine the stability of nanoparticles after the freeze-drying process. Therefore, it is key to investigate the stability of nanoparticles. Researchers have developed several methods for the production of polymeric nanoparticles. This study uses the Nanoprecipitation method to produce PLGA-PEG NPs. It is also known as the solvent displacement method. There are basic components to this method namely: PLGA-PEG (polymer), acetone (solvent) and lipophilic surfactant (coumarin-6). Characterisation of nanoparticles is crucial in order to understand their properties. Malvern zetasizer used dynamic light scattering (DLS) to investigate the characteristics (size(nm), Polydispersity (PDI) and zeta potential (mV)) of 100mg/ml PLGA-PEG in aqueously dispersed and freeze-dried samples. Scanning Electron Microscopy (SEM) was used to assess the morphology of NPs. Analysis of the effect of lyophilisation on nanoparticles was performed by using Differential Scanning Calorimetry (DSC) and Thermogravimetric Analysis (TGA).

**Results**

Aqueously dispersed NPs were stable over 4 weeks. Nanoparticles were found to be smooth and spherical and percent yield following lyophilisation for all formulations was high (>75%). There was significant size increase observed for NPs freeze-dried without trehalose. Size increase for samples freeze-dried with the highest trehalose content was not significant. Differential Scanning Calorimetry (DSC) showed that addition of the cryoprotectant did not affect the glass transition (Tg) state for freeze-dried NPs. In the TGA, no residual moisture present in NP samples after lyophilisation.

**Discussion**

Freeze-drying led to size increases, the addition of cryoprotectant reduced stresses leading smaller increases in size. The nanoprecipitation and freeze-drying process did not have an effect on NP thermal properties The lyophilisation procedure was efficient in removing all moisture present Nanoparticle size is critical, not only in determining the release profile and degradation behavior, but also in determining the efficacy of the therapeutic agent in terms of tissue penetration and cellular uptake.

## A37 Knowledge, attitude and practice of healthcare providers towards dengue fever, in Khartoum, Sudan, 2017

### Rawan Elhassan^1^, Isam Elkhidir^2^, Asmaa Muawia^1^, Sahar Taha^1^

#### ^1^Medicine, University of Khartoum, Khartoum, Sudan; ^2^Microbiology and Parasitology Department, Medicine, University of Khartoum, Khartoum, Sudan

**Introduction**

Dengue fever is the most common arthropod borne illness in humans. The increasing frequency of dengue epidemic worldwide calls for better understanding of the epidemiology of dengue infection with regards to the susceptibility of African population.

**Methods**

A cross sectional study.

**Results**

Among participants 42.4% were male, 57.6% female, 71.5% below 30 years, 32.6% are house officers. Regarding knowledge of dengue fever, 25% did not know what dengue fever is, 62% knew the correct method of transmission, 68.8% knew when to suspect dengue, 46.5% did not know how to give advice on prevention of dengue, 30.2% knew WHO guidelines for dengue. 5.6% did not know the indication for hospitalization. 56.9% had wrong answers regarding dengue management. Regarding attitude towards dengue among 63.9% who knew the correct attitudes, 80.4% didn't have the adequate resources to treat patients. 30.6% believe that they are not fully trained to manage dengue without warning signs. Regarding practice among 72.9%, 56.9% did not receive training in diagnosis or treating dengue fever.

**Discussion**

The study concluded that 75% of Healthcare providers knew dengue fever, but 62% knew the correct method of transmission and only 13.2% were familiar with WHO guidelines.

## A38 Effect of acupuncture as therapy in Bell’s palsy of facial nerve

### Sandro Kalember, Filip Katanić, Aleksandar Kopitović, Svetlana Simić

#### Department of Neurology, Faculty of Medicine, University of Novi Sad, Novi Sad, Serbia

**Introduction**

Idiopathic facial paralysis (Bell’s palsy) is one of the most common pathologies that affects the facial nerve. Exact etiology and the best therapy for Bell’s palsy are yet to be discovered.

**Methods**

This prospective study consisted of 40 patients with Bell’s palsy who were examined at beginning and at the end of acupuncture treatment. Severity of palsy was measured using Sunnybrook (SB) and House-Brackmann (HB) scale, while single muscles were tracked with scale for measuring muscle strength, before and after treatment. We measured percentage of patients with complete recovery (HB 1; Sb>91) after treatment, and we compared it between men and women and left/right Bell’s palsy.

**Results**

After three months and 30 acupuncture treatments, complete recovery was recorded at 45.5% (acc. to HB 1) and 40% (acc. to Sb>91). According to the Sb scale progress is statistically significant between men and women, p<0.05; Scales for single facial muscle strength have shown that there is a difference in progress of two muscles: m. quadratus labii superiors and m. depressor anguli oris. There is no statistical significance between left and right Bell’s palsy.

**Discussion**

The aim of our study was to prove acupuncture’s effect in treatment of Bell’s palsy. Our other goals were comparing acupuncture effect between genders and left/right Bell’s palsy.

## A39 Retrospective demographic review on patients recruited for the paternal sperm epigenome study

### Amy Pawson^1^, Karen Lockyear^2^, Sergey Moskovtsev^2,3^, Sarah Kimmins^6^, Linda Dodds^7^, Hope Weiler^8^, Clifford Librach^2,3,4,5^

#### ^1^School of Medicine, Royal College of Surgeons, Dublin, Ireland; ^2^Create Fertility Centre, Toronto, Canada; ^3^Department of Obstetrics and Gynecology, University of Toronto, Canada; ^4^Department of Physiology, University of Toronto, Toronto, Canada; ^5^Department of Gynecology, Women’s College Hospital, Toronto, Canada; ^6^Department of Animal Science, McGill University, Montreal, Canada; ^7^Perinatal Epidemiology Research Unit, Dalhousie University, Halifax, Canada; ^8^School of Dietetics and Human Nutrition, McGill University, Montreal, Canada

**Introduction**

Infertility is a global problem that appears to be on the rise. It is documented that 15% of couples of reproductive age suffer from this issue, with 25% of cases being due to male factors. Researchers have indicated that diet, age, and Body Mass Index (BMI) are considered possible reasons for male factor infertility due to their effects on semen status. The purpose of this review is to support the ongoing study examining the effects of paternal nutritional status and adiposity on the sperm epigenome as well as transgenerational inheritance.

**Methods**

A retrospective chart review was conducted and clinical parameters were collected from 148 charts. The following data was extracted: age, education, life style factors, health characteristics, semen analysis results, and pregnancy outcomes.

**Results**

In total there were 148 charts reviewed. The average age of males seeking fertility treatment is 38, females, 36. 79% of males and females experience primary infertility. The rate of alcohol consumption, recreational drug use, and smoking is under 10%. The average BMI of males seeking treatment is 27.4. The semen analyses are normal. 75% of couples are treated within the first 6 months of being seen at the clinic. ICSI/IVF has the highest success rate (25%) among couples.

**Discussion**

Analyzing the data extracted from patient charts gives a detailed description of the type of population seeking fertility treatment. This study is currently ongoing and the data collected from this retrospective review will be used to enhance understanding of the sperm epigenome.

## A40 Impact of universal newborn hearing screening (UNHS) on cochlear implanted children in Ireland

### Melissa Mae Gabriel^1^, Christine McHugh^2^, Jyoti Thapa^2^, Fergal Glynn^2^, Peter Walshe^2^, Cristina Simeos-Franklin^2,3^, Laura Viani^1,2,3^

#### ^1^Medicine, Royal College of Surgeons in Ireland, Dublin, Ireland; ^2^National Cochlear Implant Programme, Beaumont Hospital, Dublin, Ireland; ^3^School of Medicine, Trinity College, Dublin, Ireland

**Introduction**

Cochlear Implantation (CI) is an established treatment for severe to profound hearing loss (HL). In April 2011, Universal Newborn Hearing Screening (UNHS) was implemented in Ireland. This research aimed to evaluate the CI clinical pathway for UNHS referrals and compare functional outcomes between UNHS and Pre-UNHS referrals.

**Methods**

Implanted children referred to the National Cochlear Implant Programme (NCIP) via UNHS from November 2011 to December 2016 were categorised based on general health. Average times between the main milestones in the CI clinical pathway were evaluated. In the second study, functional outcomes, which had been assessed using Categories of Auditory Performance (CAP) and Speech Intelligibility Rating (SIR) were compared between implanted children with no additional needs referred via UNHS and those referred Pre-UNHS under the age of four, from January 2005 to June 2011. Factors investigated were age at referral and age at CI.

**Results**

Mean time taken in the CI clinical pathway for UNHS children with no additional needs was 34% shorter than that for complex children. Their primary delay was in having pre-implant imaging while the latter faced challenges in obtaining pre-operative clearance. Implanted children with no additional needs referred via UNHS were both referred and implanted at a younger age than those referred Pre-UNHS. They also achieved better CAP and SIR scores 2 years post-implant.

**Discussion**

UNHS in Ireland is crucial for earlier diagnosis and intervention of congenital HL, as there is a sensitive period for sensory auditory development. These translate to a positive impact on functional outcomes in children.

## A41 The role of FKBPL in vascular dysfunction and inflammatory disease

### Bharti Kewlani^1^, Tracy Robson^2^, Stephanie Annett^2^

#### ^1^Medicine, Royal College of Surgeons in Ireland, Dublin, Ireland; ^2^Department of Molecular and Cellular Therapeutics, Royal College of Surgeons in Ireland, Dublin, Ireland

**Introduction**

FK506 Binding Protein Like (FKBPL) is a novel member of the immunophilin protein family. It targets the cell surface receptor, CD44, disrupting tumour angiogenesis and cancer stem cells, resulting in significant growth inhibition. AD-01 is a novel therapeutic based on the active domain of FKBPL. Previous studies noted that FKBPL deficient mice demonstrate enhanced angiogenesis and increased inflammation. This project examined the role of FKBPL and AD-01 on endothelial barrier function and pro-inflammatory cytokine release, both of which play an integral role in the pathogenesis of conditions such as stroke, insulin-resistant diabetes and septic shock.

**Methods**

THP-1 cells (monocytes) were stimulated with LPS (lipopolysaccharide) ± AD-01, and ELISA was used to determine IL-1β, IL-6 and TNF-α cytokine secretion. siFKBPL knockdown transfection was performed on HMEC (endothelial) cells and the Evans blue vascular permeability assay was employed to assess effects of FKBPL on endothelial permeability. Finally, Western blotting was used to evaluate the effects of AD-01 on the MAP-K, STAT3 and NF-κB intracellular signaling pathways.

**Results**

AD-01 significantly abrogated LPS-induced NFĸB and STAT3 activation and Il-1β secretion. Furthermore, siRNA mediated knockdown of FKBPL resulted in a highly significant increase in vascular permeability which was partially reversed by treatment with AD-01.

**Discussion**

FKBPL and AD-01 demonstrate a clear role in both response to LPS signaling and protecting vascular integrity. FKBPL’s therapeutic peptide, AD-01, could therefore be useful in the treatment of diseases where inflammatory responses mediated by high IL-1β secretion are implicated.

## A42 Describing the sexual and vaginal health of hormone-sensitive breast cancer survivors on maintenance anti-estrogen therapy at St. Michael’s Hospital

### Nadine Madani, Christine Brezden-Masley, Amy Skitch

#### Hematology-Oncology Clinical Research Group, St. Michael’s Hospital, Toronto, Canada

**Introduction**

70% of breast cancer (BC) cases are hormone-sensitive positive (HR+) breast cancers, expressing either or both estrogen (ER) and progesterone receptors (PR). Anti-estrogen endocrine therapies targeting ER pathways are the first line of treatment. Tamoxifen, a Selective Estrogen-Receptor Modulator, is indicated for early ER+ tumours, while third generation aromatase inhibitors are the standard treatment for post-menopausal women. Although effective, endocrine therapies cause a host of side effects (SEs) including menopausal symptoms, and vaginal pathologies due to diminished estrogen levels. Side effects advance to cause body image dysmorphia, lower quality-of-life (QoL), and psychological stress. We conducted a needs-assessment study to investigate the prevalence of vaginal toxicities and sexual dysfunction in these patients, and how these affect patients’ QoL secondarily.

**Methods**

A comprehensive anonymous survey was developed for HR+ BC patients at St. Michael’s Hospital’s oncology clinic who have been on maintenance anti-estrogen therapy for 6 months minimum. The survey includes validated questionnaires, and examines patients’ treatment history, side effects, sexual health, and interactions with treating oncologists.

**Results**

Of 48 patients surveyed, 67% have not been asked about vaginal or sexual SEs by their oncologists. Chronic vaginal dryness: 62%, abnormal vaginal discharge: 65%, dyspareunia: 33%, depression: 65%. 35% requested to be referred to a specialist. 83% requested they be informed of management options.

**Discussion**

Majority of patients surveyed report QoL-limiting SEs that can be managed if addressed. Results underscore what should be addressed by oncologists that is often not, and is vital to providing wholesome BC treatment.

## A43 Co-morbidities, co-medications, and concordance in breast cancer survivors: A prospective cohort study

### Weng Kit Chan^1^, Kathleen Bennett^2^, Amelia Smith^3^

#### ^1^Medicine, Royal College of Surgeons in Ireland, Dublin, Ireland; ^2^Division of Population Health Sciences, Royal College of Surgeons in Ireland, Dublin, Ireland; ^3^Pharmacology and Therapeutics, Trinity College Dublin, Dublin, Ireland

**Introduction**

Breast cancer is the most prevalent cancer among women worldwide, with 1.7 million diagnosed in 2012. In Ireland, there is little information on the extent of co-morbidities and co-medications in breast cancer survivors. This study aims to examine: (1) the prevalence of co-morbidities compared with a general Irish female population (TILDA), (2) the prevalence of co-medications and (3) co-medications as a proxy for disease in an Irish national breast cancer cohort.

**Methods**

There were n=974 women diagnosed with breast cancer consenting to a self-completed questionnaire (July 2014 - Aug 2017), recruited from five hospitals (Cork University, Beaumont, Mid-Western Regional, Galway University, and St James’s).

**Results**

The most prevalent co-morbidity was hypertension (27.1%, total n=844), followed by arthritis (13.9%, total n=828), and asthma (9.1%, total n=831). Prevalence of co-morbidities in this cohort was significantly lower compared to a general population (TILDA), of the same ages. 77.3% (n=752) of female breast cancer survivors were on 0-4 co-medications. The remainder (22.7%, n=222) had polypharmacy (5+ co-medications). The most prevalent co-medication was alimentary tract drugs (38.2%, n=372), followed by nervous system drugs (32.8%, n=319), and cardiovascular drugs (27.9%, n=272). There was significant discordance between CVD drugs and reporting of Heart Disease; similarly for respiratory drugs and reporting of Asthma/COPD (p<0.001).

**Discussion**

The significantly lower prevalence of reported co-morbidities in this breast cancer cohort compared to TILDA is of interest. The lack of agreement between selected co-morbidities and co-medications is likely due to under-reporting in breast cancer. Further studies would be helpful to replicate our findings.

## A44 Examining online dialogue of K-12 sex education in the 2015 Ontario health & physical education curriculum: Towards collaborative course construction

### Yusuf Ahmed^1^, Joyce Nyhof-Young^2^, Xiaohe (Diana) Sun^2^, Karissa Holyer^2^, Ada Posner^2^

#### ^1^Faculty of Sciences, University of Western Ontario, London, Canada; ^2^University of Toronto, Family and Community Medicine, Toronto, Canada

**Introduction**

Technology has revolutionized how youth communicate and receive sexual content. 25% of Canadian high school students report having had sex, and 25% of those do not use condoms. Sex education (SE) can delay onset of sex, promote contraceptive use, and reduce teen pregnancy and STI rates. In February 2015, the Ontario government released an updated K-12 Health & Physical Education curriculum. SE, 10% of this curriculum, has elicited strong public debate. We explored online stakeholder perspectives to understand issues and stances and inform ongoing SE delivery.

**Methods**

Online English publications (January 2010 - June 2017) were selected from 100 top article hits for each key phrase (e.g. “Ontario sex ed” and “Ontario health and physical education”) searched on Google, Canadian Newsstream, and CPI.Q., duplicate hits eliminated, a coding framework constructed and descriptive thematic analysis performed by 3 coders.

**Results**

674 articles were abstracted and categorization begun (i.e. government, news, personal, social organization). Preliminary analysis of 12 articles (3 per category) was used to refine our coding frame. Diverse stakeholder stances (parents, educators, politicians, healthcare/religious providers, etc.) were captured. 8/12 supported the new curriculum, praising updated topics (e.g. LGBTQ2S, consent, cyber-safety). 3/12 articles opposed reform (e.g. rejecting LGBTQ2 and early sexuality content, state encroachment on family, cultural and religious values).

**Discussion**

This study highlights themes of curriculum rationales, values, strengths, apprehensions, and misconceptions of the SE curriculum by stakeholders. Enhanced understanding of stakeholders’ positions aims to improve collaboration between stakeholders all working to support the health and wellbeing of Ontario’s children and youth.

## A45 Pilot study on the heritability of mean body temperature and its correlation to frailty

### Tan Tx Rachel^1^, Steve CJ^2^

#### ^1^Medicine, Royal College of Surgeons in Ireland, Dublin, Ireland; ^2^Department of Twin Research & Genetic Epidemiology, Kings College London, London, United Kingdom

**Introduction**

Older and frail adult tends to have lower core body temperature, and it can be an indicator of early mortality marker. A few animal studies demonstrate mean body temperature as a heritable trait. The aim is to study if there is a relationship between temperature and frailty and estimate to what extend that temperature is heritable in humans.

**Methods**

The study consists of twins and non-twins between the ages of 22-78 years old. 57 twin participants and 19 non-twin participants are recruited in this study. Mean body temperature (MTB) were used to estimate the variation with age. Frailty were assessed through 4m-walk test (4MWT) and frailty index. All datas were statistically analysed through STATA 13. A linear regression scatterplot used to analyze the association of temperature with age. Multivariate regression model on age, gait speed, BMI and Frailty index to explore how they were related temperature. Heritability estimate for temperature in twins were derived through intraclass correlation coefficient, calculated by falconer’s formula.

**Results**

The result shows consistent decrease in MTB in older age individual and was the only independent variable that statistically significant and correlated to temperature. The hypothesis of relationship between temperature and frailty was incorrect. However, heritability estimate for temperature were statistically significant in monozygotic twins

**Discussion**

This study also gave a novel evidence that mean body temperature is a heritable phenotype in human to a certain extend, and give us potential directions for future research in this area.

## A46 Dose requirements of intravenous enoxaparin in critically ill patients

### Rahin Wahedi^1^, Michael Tryba^2^

#### ^1^Faculty of Medicine, University of Southampton, Southampton, United Kingdom; ^2^Klinikum Kassel-Gesundheit Nordhessen, Department of Anaesthesiology, Intensive Care and Pain Therapy, Kassel, Germany

**Introduction**

Enoxaparin is administered for the prophylaxis of venous thromboembolism (VTE). Its effectiveness is determined using anti-Xa levels. Based on literature, the standard dose of 40 mg subcutaneously yields sub-therapeutic anti-Xa levels in 60-80% of surgical intensive care unit (ICU) patients. Since April 2015, enoxaparin is given intravenously in surgical ICU at the Klinikum Kassel.

**Methods**

64 surgical ICU patients aged ≥18, who received intravenous enoxaparin for ≥7 days, were retrospectively analysed. The percentage of patients below, in and above therapeutic anti-Xa range was calculated for each treatment day. The aim is to investigate how often anti-Xa levels were in the therapeutic range (0.1-0.3 IU/ml) and also to record resulting thromboembolic and bleeding complications.

**Results**

The percentage of patients within therapeutic anti-Xa range increased from treatment day 4 to 7 from 17.2% to 40% and on days 16, 19 and 22 from 37.5% to 50% to 71.4%, respectively, with 71.4% on treatment day 22 being the peak. Multiple dose increases correlated with high noradrenaline doses (≥1 mg/h) and sepsis. Dose requirements of ≥120 mg enoxaparin were found in patients with high noradrenaline doses. 1 case of VTE and 1 bleeding episode were recorded in this study.

**Discussion**

81.3% of patients achieved sub-therapeutic anti-Xa levels on day 4, suggesting the initial dose of 40 mg to be inadequate. However, only 1 thromboembolic event was recorded. Before comparing the clinical efficacy of subcutaneous and intravenous enoxaparin, further studies involving higher initial doses, faster dose increases and daily anti-Xa-measurements are required.

## A47 Outcomes of radical treatment of NET liver metastases: A single tertiary centre 18-year experience

### Amanda Tan^1^, Brian Davidson^2^, James Pape^2^, Panagis Lykoudis^2^

#### ^1^NIL, Royal College of Surgeons in Ireland, Dublin, Ireland; ^2^Department of Hepato-Pancreato-Biliary Surgery & Liver Transplantation, Royal Free Hospital, London, United Kingdom

**Introduction**

The presence of liver metastases is a poor prognostic factor in patients with Neuroendocrine Tumours (NET). Resection of NET liver metastases (NET mets) has been reported to be associated with good long-term outcomes but must be balanced against the risks of major surgery. Thermal ablation or arterial embolisation offers an alternative to surgery. We aim to review the radical treatment outcomes of NET mets.

**Methods**

Data was collected retrospectively between 1998 to 2016 of consecutive patients with NET mets who underwent radical treatment (surgical resection, radiofrequency ablation (RFA) and/or trans-arterial embolisation (TAE)) in a single specialist HPB/NET centre.

**Results**

Fifty-four patients (38.9% male) were included. Median age at treatment was 55 years. Twenty-one patients had a pancreatic primary NET, 24 in the midgut and 9 in other locations. Forty-four patients had a previous operation for primary tumor resection. Radical therapy consisted of surgical resection in 41, TAE in 5, RFA in 2 and 1 underwent liver transplant. Five underwent surgical resection and simultaneous intra-operative RFA. Those with high-grade tumour (n = 8) had a significantly shorter survival. Median follow-up period was 49 months. Progression occurred in 50%. Median progression-free survival was 14.5 months. Forty patients were alive at last follow-up. Median overall survival was 41 months.

**Discussion**

In this single-centre experience, liver resection has been the main form of radical therapy for NET mets despite RFA and TAE being available. Although progression occurred in 50%, 74% of patients were alive at median follow-up of over 4 years supporting a radical approach for selected patients with NET mets.

## A48 Ex-vivo gene therapy in mouse model of hereditary tyrosinemia type 1

### Sowmya Sharma, Raymond Hickey, Caitlin VanLith, Rebekah Guthman

#### Department of Surgery, Department of Molecular Medicine, Mayo Clinic, Rochester, Minnesota, USA

**Introduction**

Hereditary Tyrosinemia Type 1 (HT1) is a rare disorder caused by mutation of ‘FAH’ enzyme in the tyrosine degradation pathway, resulting in accumulation of toxic substrates which cause liver and kidney disease. Current management options are limited and rarely successful. The genetic root of the disease makes gene therapy an attractive alternative, allowing correction of the causative mutation. CRISPR-Cas9 is an endonuclease system used for correction of genetic mutations. This project aimed to demonstrate concept of curative HT1 treatment by ex-vivo mutated ‘Fah’ gene correction in mouse models by CRISPR-Cas9 system, delivered via viral vectors.

**Methods**

Adeno-associated virus (AAV) vectors were selected to deliver CRISPR-Cas9 system into mouse hepatocytes. Genetically modified mice with mutated Fah gene were used for the study, as per IACUC animal research standards. Hepatocytes isolated from these mice were transduced with AAV vectors ex-vivo, and transplanted into mice livers via the portal system by splenic injections.

**Results**

For several months following the hepatocyte transplantation, the mice were monitored and cycled on and off medication to allow hepatocyte growth, allowed by the inherent regenerative ability of hepatocytes. These mice were then sacrificed and liver biopsy and staining was done to check for presence of FAH enzyme, which showed areas of ‘FAH’ positive cells.

**Discussion**

Gene therapy can be used to restore normal enzyme production in mutated hepatocytes, which provides an alternative curative option to a disease which was previously only symptomatically managed. This project establishes proof of the concept for using CRISPR-Cas9 delivered via viral vectors to achieve this.

## A49 Monitoring cancer progression in metastatic pancreatic cancer patients using cell-free DNA (cfDNA) concentrations

### Emily Hutchings^1^, Trevor Pugh^1^, Tiantian Li^1^, Olena Kis^1^, Anna Dodd^2^, Rene Quevedo^1^, Sean Creighton^2^, Tingting Wang^1^, Tracy Zhang^3^, Scott Bratman^1^, Jennifer Knox^2^, Kyaw Aung^4^

#### ^1^Department of Medical Biophysics, Princess Margaret Cancer Centre, University Health Network, University of Toronto, Toronto, Canada; ^2^Department of Medical Oncology, Princess Margaret Cancer Centre, University Health Network, University of Toronto, Toronto, Canada; ^3^Cancer Genomics Program, Princess Margaret Cancer Centre, University Health Network, University of Toronto, Toronto, Canada; ^4^Department of Medical Oncology and Hematology, Princess Margaret Cancer Centre, University Health Network, University of Toronto, Toronto, Canada

**Introduction**

Pancreatic cancer has a high mortality rate and biopsies are highly invasive. Blood plasma of cancer patients contains circulating tumour DNA (ctDNA), within total cell-free DNA (cfDNA) measures. ctDNA has been demonstrated to track cancer progression in multiple malignancies. If ctDNA can be detected in pancreatic cancer patients, it may be used in diagnosis and treatment monitoring. This study evaluates the feasibility of using cfDNA to measure metastatic pancreatic cancer burden.

**Methods**

Whole blood samples (40 ml) were collected from 15 patients across multiple time points. Total cfDNA was extracted from blood plasma using QIAamp Circulating Nucleic Acid Kit and quantified using Qubit. Agilent TapesStation was used to assess the size distribution of DNA fragments to detect genomic DNA contamination. Total cfDNA concentrations were compared to clinical markers of pancreatic tumour burden, including CA19-9 and measured tumour size, using R programming.

**Results**

Measurable quantities of cfDNA were isolated from all blood samples analyzed in this study. However, the relative concentration of cfDNA in plasma did not correlate with pancreatic tumour burden, indicating that cfDNA is not an accurate tool for monitoring cancer progression.

**Discussion**

Plasma concentrations of cfDNA do not accurately represent pancreatic cancer progression. Variability in the proportion of normal and ctDNA in cfDNA samples may contribute to inconsistencies between cfDNA and tumor burden. Future direction will be to evaluate ctDNA levels from cfDNA using next generation sequencing. Plasma concentrations of ctDNA will be compared with known tumour burden to assess the reliability of ctDNA as a biomarker in pancreatic cancer.

## A50 Evaluation of the effects of a Pinus Brutia bark extract on serum lipid profile and oxidative stress in an experimental arterial hypertension model

### Nicoleta Dumitrescu^1^, Manuela Ciocoiu^2^, Alexandru Costache^2^, Stefania Duca^2^, Amalia Ilasoaia^2^

#### ^1^Faculty of Medicine, “Grigore T. Popa” University of Medicine and Pharmacy Iasi, Iasi, Romania; ^2^Department of Physiopathology, “Grigore T. Popa” University of Medicine and Pharmacy Iasi, Iasi, Romania

**Introduction**

New antihypertensive therapies offering an improvement of the oxidative mechanisms involved in the development of arterial hypertension (AHT) would be of great practical value. The aim of our study was to investigate the effects of Pinus brutia bark extract (PbE) on serum lipid profiles and oxidative stress in N(G)-Nitro-L-arginine-methyl ester (L-NAME)-induced hypertension.

**Methods**

We conducted an 8-week experiment on 48 white male Wistar rats distributed in 4 groups, as follows: (i). Group W - control animals; (ii) Group PbE - animals received Pinus brutia extract ( 39,38 ± 0,24 mg/g/day); (iii) Group AHT - animals treated with L-NAME 40 mg/kg/day; (iv) Group PbE+AHT – animals received simultaneously PbE+L-NAME. For each animal, we recorded blood pressure, heart beats, serum lipid profiles, and total antioxidant capacity (TAC).

**Results**

After 8 weeks, L-NAME increased diastolic blood pressure by 19.35% over Group W, but in Group EpB+L-NAME diastolic blood pressure increased only by 5.07%. Both Group PbE and Group PbE+L-NAME showed decreased cholesterol values compared with Group W (65,10±5,08 vs 71,70±3,77), and Group AHT (69,20±5,88 vs 71,70±3,77) respectively. Both Group PbE and Group PbE+L-NAME showed an increase of high-density lipoprotein cholesterol values compared with Group W (33,40±4,52 vs 30,30±4,98), and Group AHT (24,2±3,35 vs 18,70±4,39) respectively. Group AHT showed significantly decreased values of TAC (1,133 mmol/L), but concomitant administration of PbE+L-NAME diminished the overall antioxidant status (1,236 mmol/L).

**Discussion**

Our experiment demonstrated that PbE improved lipid profile and reduced pro-oxidative effects of L-NAME, thus suggesting a possible role of the extract in the management of AHT.

## A51 Assessing role of SATB2 and SATB2-AS1 genes in colorectal cancer

### ZI Yan Chiah^1^, Darran O'Connor^2^, Camille Hurley^2^, Ian Miller^3^, Annette Byrne^3^, Sudipto Das^2^

#### ^1^RCSI Research Summer School, Royal College of Surgeons in Ireland, Dublin, Ireland; ^2^Department of Molecular and Cellular Therapeutics, Royal College of Surgeons in Ireland, Dublin, Ireland; ^3^Department of Physiology and Medical Physics, Royal College of Surgeons in Ireland, Dublin, Ireland

**Introduction**

SATB2-AS1 is an antisense transcript to SATB2, the latter is described as a well categorized tumour suppressor gene in colorectal cancer (CRC). Thus, it is plausible that SATB2-AS1 might play a role in CRC progression. This study was aimed to examine the expression levels of SATB2 and SATB2-AS1 both in vitro and in vivo.

**Methods**

mRNA expression of SATB2 and SATB2-AS1 was assessed in SW620 (metastatic) and SW480 (non-metastatic) CRC cell line and in vivo orthotopic mice models (same cell line) treated with either FOLFOX alone, combination of FOLFOX and Avastin, Avastin alone or vehicle. RNA was extracted and used for cDNA synthesis followed by gene specific expression testing using quantitative real-time PCR (qRT-PCR).

**Results**

Endogenous levels of both SATB2 and SATB2-AS1 were higher in SW480 compared to SW620 cell line. SATB2 was elevated in SW620 combination treated samples, in SW480 FOLFOX alone treated sample and was lowered in SW480 combination treated samples in mice models relative to vehicle. Moreover, SATB2-AS1 was up-regulated in SW620 Avastin alone treated samples and in SW480 combination treated samples in derived mouse model relative to the vehicle. We also examined the potential silencing of the SW620 genes due to DNA methylation (5’-aza-2’-deoxycytidine treatment) compared to SW480. No significant alteration was found but both genes were significantly elevated relative to vehicle.

**Discussion**

Elevated SATB2 and SATB2-AS1 levels suggest a plausible protective role of both genes in CRC. In addition, we demonstrate that both SATB2 and SATB2-AS1 appear to be co-regulated by DNA methylation. Future studies will allow us to ascertain the precise role of this anti-sense transcript in CRC.

## A52 The effect of DNA damage repair pathway inhibition on neuronal survival and regeneration in the spinal cord

### Kate Alexander, Zubair Ahmed, Sarina Surey

#### Institute of Inflammation and Ageing, University of Birmingham, Birmingham, UK

**Introduction**

Spinal cord injury is a highly disabling, permanent condition. In theory, promoting neuronal regeneration in a damaged spinal cord could allow connections between severed neurons to repair resulting in restoration of function. This study aimed to test the effect of DNA damage repair pathway (DDRP) inhibition on neuronal survival and neurite outgrowth.

**Methods**

Inhibition of the DDRP was achieved by inhibiting the Mre11-Rad50-Nbs1 (MRN) complex. Four different MRN inhibitors (mirin, peptide 3, I2, A2) were applied to rat dorsal root ganglion cultures at varying concentrations for 3 days (n= 3 for each treatment). Mean number of surviving neurons, number of neurons with neurite growth and average maximum length of neurites was determined for each treatment. Statistical analysis involved one-way ANOVA followed by Bonferroni post-hoc testing.

**Results**

Treatment with peptide 3 (100nM) promoted all measures. Mirin (10μM) only significantly promoted survival. A2 and I2 (10μM) resulted in poor survival and no significant outgrowth; chemical assays showed that these two treatments resulted in complete MRN inhibition.

**Discussion**

Results regarding peptide 3 suggest that DDRP inhibition via MRN inhibition can promote neuronal survival and outgrowth in rat dorsal root ganglion neurons. However, results with A2/I2 suggest that complete inhibition of MRN is unfavourable. Further studies are required to determine the optimum level of MRN inhibition for maximum dorsal root ganglion survival and growth. Although this is a preliminary study, overall, the results suggest that DDRP inhibition has capacity to promote neuronal survival and outgrowth; therefore could have future potential in spinal cord injury treatment.

## A53 Oral vitamin B12 replacement in Crohn's disease with terminal ileal disease

### Richard Bresler^1^, Ian Plener^2^

#### ^1^Royal College of Surgeons in Ireland, Dublin, Ireland; ^2^Division of Gastroenterology, University of Toronto, Ontario, Canada

**Introduction**

Vitamin B12 deficiency is common in Crohn’s disease, particularly in patients with small bowel disease. While vitamin B12 replacement has traditionally been achieved by parenteral therapy, evidence in the general population suggests that oral replacement therapy may be adequate for most patients.

**Methods**

Patients with Crohn's disease and concomitant vitamin B12 deficiency who were attending a specialist clinic were identified. Patients newly vitamin B12-deficient (Category 1), those predicted to develop B12 deficiency, and patients already on parenteral B12 (Category 2) were transitioned to oral vitamin B12 replacement therapy (1200mcg daily). Levels of serum vitamin B12 and baseline hematology values were recorded.

**Results**

A total of 44 patients are included. All patients in the follow-up period achieved or maintained normal B12 levels with oral therapy. In category 1 patients, the median B12 level at baseline was 142 (IQR: 130-150), increasing to 290 (IQR: 233-387) at 6 months and 350 (IQR: 280-499) at 12 months. Their B12 levels remained normal throughout the follow-up period, with the median of 440 (IQR: 340-619). In category 2 patients, the median B12 level at baseline was 401 (IQR: 351-646), at 6 months 460 (IQR: 382-557), and at 12 months 475 (IQR: 409-601). Their B12 level remained above normal throughout the follow up period (median of 680 (IQR: 504-779)).

**Discussion**

Oral vitamin B12 replacement therapy was effective and well-tolerated in this group of patients with Crohn’s disease.

## A54 An inflammatory biomarker study of psychosis: A longitudinal study in an at risk population

### Mohammed Alghamdi, David Cotter, Sophie Sabherwal

#### School of Medicine, Royal College of Surgeons Ireland, Dublin, Ireland

**Introduction**

Biomarkers are a important diagnostic tool in medicine, as they aid in early detection paving the way for screening, early treatment and acquiring knowledge about the disease. Many blood biomarkers have been identified in schizophrenia, bipolar disorder and major depressive disorder. In this study, we aim to identify protein biomarkers for psychosis in blood, specifically apolipoproteins.

**Methods**

The plasma samples used in this study were taken from the UK Avon Longitudinal Study of Parents and Children (ALSPAC) cohort study at ages 12 and 18, samples at age 12 were divided into 3 groups: no psychotic experiences, present psychotic experiences and psychotic disorder. Samples were put through High Pressure Liquid Chromatography (HPLC) and analysed using Tandem Mass Spectrometry (MS-MS). Data from spectrometry was then processed through Skyline proteomics analysis program using a Data Independent Acquisition (DIA) technique and standardized using Pierce Peptide. Data from the third patient group included both control and At Risk Metal State (ARMS).

**Results**

The target list that was analysed using Skyline included 13 apolipoproteins. An increased expression of targets was observed in ARMS groups and from this target list, Apolipoprotein B (p=0.28) and Apolipoprotein E (p=0.12) were the most promising with the lowest p values after statistical analysis but none of the results showed significance.

**Discussion**

Results of this study show feasibility for early detection of psychosis in psychological disorders and opens the door for further investigation in the field with other possible biomarkers. Further work is warranted to find future potential candidates as biomarkers that can be used as diagnostic tools in detection of psychosis.

